# Encephalopathy induced by Alzheimer brain inoculation in a non-human primate

**DOI:** 10.1186/s40478-019-0771-x

**Published:** 2019-09-04

**Authors:** Charlotte Gary, Suzanne Lam, Anne-Sophie Hérard, James E. Koch, Fanny Petit, Pauline Gipchtein, Stephen J. Sawiak, Raphaëlle Caillierez, Sabiha Eddarkaoui, Morvane Colin, Fabienne Aujard, Jean-Philippe Deslys, French Neuropathology Network, Charles Duyckaerts, Véronique Sazdovitch, Sabrina Leclère-Turbant, Marie-Claire Artaud-Botté, Anne Vital, Françoise Chapon, Jean-Louis Kemeny, Claude-Alain Maurage, Vincent Deramecourt, David Meyronet, Nathalie Streichenberger, André Maues de Paula, Valérie Rigau, Fanny Vandenbos-Burel, Danielle Seilhean, Serge Milin, Dan Christian Chiforeanu, Annie Laquerrière, Béatrice Lannes, Marie-Bernadette Delisle, Emmanuelle Uro-Coste, Emmanuel Brouillet, Luc Buée, Emmanuel E. Comoy, Fabien Pifferi, Jean-Luc Picq, Marc Dhenain

**Affiliations:** 1 Centre National de la Recherche Scientifique (CNRS), Université Paris-Sud, Université Paris-Saclay UMR 9199, Laboratoire des Maladies Neurodégénératives, 18 Route du Panorama, F-92265 Fontenay-aux-Roses, France; 2 Commissariat à l’Energie Atomique et aux Energies Alternatives (CEA), Direction de la Recherche Fondamentale (DRF), Institut François Jacob, MIRCen, 18 Route du Panorama, F-92265 Fontenay-aux-Roses, France; 3 University of Wisconsin Oshkosh, 800 Algoma Boulevard, Oshkosh, WI 54901 USA; 4 Wolfson Brain Imaging Centre, University of Cambridge, Addenbrooke’s Hospital, Cambridge Biomedical Campus, Cambridge, CB2 0QQ UK; 5 Behavioural and Clinical Neuroscience Institute, University of Cambridge, Cambridge Biomedical Campus, Cambridge, CB2 0QQ UK; 6 LabEx DISTALZ (Development of Innovative Strategies for a Transdisciplinary approach to ALZheimer’s disease), Université de Lille, Inserm, CHU-Lille, UMR-S1172, Alzheimer & Tauopathies, Rue Polonovski, 59045 Lille, France; 7 UMR7179 CNRS-MNHN, MECADEV (Adaptive Mechanisms and Evolution), 1 Avenue du Petit Château, 91800 Brunoy, France; 8 Commissariat à l’Energie Atomique et aux Energies Alternatives (CEA), Direction de la Recherche Fondamentale (DRF), Institut François Jacob, SEPIA, Université Paris-Saclay, 18 Route du Panorama, F-92265 Fontenay-aux-Roses, France; 9 GIE Neuro-CEB/Neuropathologist Network: Plate-Forme de Ressources Biologiques, Bâtiment Roger Baillet, Hôpital de la Pitié-Salpêtrière, 47-83 Boulevard de l’Hôpital, Cedex 13, 75651 Paris, France; 10 Laboratoire de Psychopathologie et de Neuropsychologie, EA, 2027, Université Paris 8, St-Denis, France

**Keywords:** Alzheimer’s disease, β-Amyloid pathology, Cerebral atrophy, Cognitive impairment, *Microcebus murinus*, Mouse, Neurodegenerative disease, Neuronal function, Prion, Tau pathology

## Abstract

Alzheimer’s disease is characterized by cognitive alterations, cerebral atrophy and neuropathological lesions including neuronal loss, accumulation of misfolded and aggregated β-amyloid peptides (Aβ) and tau proteins. Iatrogenic induction of Aβ is suspected in patients exposed to pituitary-derived hormones, dural grafts, or surgical instruments, presumably contaminated with Aβ. Induction of Aβ and tau lesions has been demonstrated in transgenic mice after contamination with Alzheimer’s disease brain homogenates, with very limited functional consequences. Unlike rodents, primates naturally express Aβ or tau under normal conditions and attempts to transmit Alzheimer pathology to primates have been made for decades. However, none of earlier studies performed any detailed functional assessments. For the first time we demonstrate long term memory and learning impairments in a non-human primate (*Microcebus murinus*) following intracerebral injections with Alzheimer human brain extracts. Animals inoculated with Alzheimer brain homogenates displayed progressive cognitive impairments (clinical tests assessing cognitive and motor functions), modifications of neuronal activity (detected by electroencephalography), widespread and progressive cerebral atrophy (in vivo MRI assessing cerebral volume loss using automated voxel-based analysis), neuronal loss in the hippocampus and entorhinal cortex (post mortem stereology). They displayed parenchymal and vascular Aβ depositions and tau lesions for some of them, in regions close to the inoculation sites. Although these lesions were sparse, they were never detected in control animals. Tau-positive animals had the lowest performances in a memory task and displayed the greatest neuronal loss. Our study is timely and important as it is the first one to highlight neuronal and clinical dysfunction following inoculation of Alzheimer’s disease brain homogenates in a primate. Clinical signs in a chronic disease such as Alzheimer take a long time to be detectable. Documentation of clinical deterioration and/or dysfunction following intracerebral inoculations with Alzheimer human brain extracts could lead to important new insights about Alzheimer initiation processes.

**Electronic supplementary material:**

The online version of this article (10.1186/s40478-019-0771-x) contains supplementary material, which is available to authorized users.

## Introduction

Alzheimer’s disease is a neurodegenerative disease characterized by cognitive alterations, cerebral atrophy [1] and neuropathological lesions including neuronal loss [2], accumulation of misfolded and aggregated β-amyloid peptide and tau proteins [3]. Patients exposed to cadaver-derived hormones, dural grafts or to surgical instruments, presumably contaminated with β-amyloid peptide (Aβ), have a higher risk of developing early-onset Aβ pathology than non-exposed subjects [4–6]. Experimental induction (or acceleration) of β-amyloidosis or tauopathy has been described in rodents after intracerebral and even peripheral contamination with pathological Aβ or tau-bearing brain homogenates [7, 8]. However, none of the long-term transmission studies with Aβ or tau-positive inocula provided evidence for pronounced cognitive decline or neurodegeneration [9]. Aged non-human primates can naturally develop β-amyloid lesions [10] and a long-term study in marmosets demonstrated induction of sparse β-amyloidosis 3.5 to 7 years post-inoculation, but there was no evidence of cognitive decline, neurodegeneration, functional Alzheimer’s disease hallmarks, or other clinical signs [11]. This calls for additional clinical analysis in primates after inoculation with Alzheimer’s disease brain homogenates.

Here, we used a multimodal approach, including non-invasive methods, to evaluate the impact of inoculation of human Alzheimer’s disease-brain homogenates on both brain function and integrity in mouse lemur primates (*Microcebus murinus*). These small primates (body length: 12 cm; weight: 60-120 g) have a maximal lifespan of 12 years in our colony [12], although longer lifespans have been reported in some breeding colonies [13, 14], and are considered to be “old” after 6 years [12]. Mouse lemurs are widely used models to study human aging [15] since they display age-related alterations of their sensorial system, motor functions, biological rhythms, and immune and endocrine systems [12]. Their cerebral aging profile is similar to that of humans, as some can display age-related cognitive alterations associated with cerebral atrophy [16]. Like humans and other non-human primates, they are genetically heterogeneous, providing a natural diversity of aging profiles. In addition, genes associated with β-amyloidosis, such as amyloid precursor protein (APP), are similar in humans and mouse lemurs [17]. They can develop intracellular deposits of APP/Aβ [18, 19], as well as β-amyloid plaques which can be detected in 25% of the animals over 8 years of age [20, 21], as well as tauopathy [21, 22]. Finally, their small size and reduced lifespan (compared to macaques) facilitates the creation of experimental cohorts to evaluate factors that modulate cerebral aging.

Human brain homogenates from Alzheimer’s disease or control patients were inoculated in the brain of 12 adult mouse lemurs. We performed longitudinal cognitive assessments, electroencephalography (EEG), and morphological magnetic resonance imaging (MRI) studies up to 18 months post-inoculation (mpi), followed by immunohistopathological examination of brain tissues. In parallel, transgenic mice were also inoculated to assess the pathological potential of our homogenates. The inoculation of Alzheimer’s disease-brain homogenates in primates induced an encephalopathy characterized by pronounced cognitive, functional, and morphological alterations, as well as neuronal loss. Most alterations were not seen 6 mpi but became evident at 12 mpi, ruling out immediate pathogenicity of the homogenates. Sparse β-amyloid and tau lesions were also detected in the brains of Alzheimer’s disease-inoculated mouse lemurs at the inoculation sites and spreading from the inoculation sites could be highlighted in some animals, but these lesions were never detected in control animals. These results show that inoculation of Alzheimer’s disease brain homogenates induces a pathology leading to cognitive impairments, clinical signs, neuronal loss and alteration of neuronal activity in a primate.

## Materials and methods

### Human brain samples

Frozen brain tissue samples (parietal cortex) from two Alzheimer’s disease patients (Braak and Braak stage VI, Thal stages 5 and 4, respectively) and one control individual (Braak and Braak/Thal stages 0) were obtained from brains collected in a brain donation program of the GIE NeuroCEB Brain Bank run by a consortium of Patient Associations: ARSEP (French association for research on multiple sclerosis), CSC (cerebellar ataxias), France Alzheimer, and France Parkinson, with the support of the Fondation Plan Alzheimer and IHU A-ICM (Additional file 1: Table S1). The consent forms were signed by either the patients themselves or their next of kin in their name, in accordance with French bioethics laws. The GIE NeuroCEB Brain Bank has been declared at the Ministry of Higher Education and Research and has received approval to distribute samples (agreement AC-2013-1887).

These brain tissues were first assessed by immunohistochemistry. They were cut into 4-μm-thick paraffin sections. Sections were deparaffinized in xylene, successively rehydrated through ethanol (100, 90, and 70%), and finally rinsed under running tap water for 10 min. They were then incubated in 99% formic acid for 5 min, washed again under running tap water, quenched for endogenous peroxidase with 3% hydrogen peroxide and 20% methanol, and finally washed in water. Sections were then blocked by incubating the sections at room temperature for 30 min in 4% bovine serum albumin (BSA) in 0.05 M tris-buffered saline, with 0.05% Tween 20, pH 8 (TBS-Tween, Sigma). They were then incubated overnight at + 4 °C with the 6F3D anti-Aβ antibody (Dako, Glostrup, Denmark, 1/200), polyclonal anti-tau antibody (Dako, Glostrup, Denmark, 1/500), and monoclonal anti-alpha-synuclein (LB509, Zymed, USA, 1/250) routinely used for the detection of β-amyloid, tau and alpha-synuclein deposits, respectively. The sections were further incubated with a biotinylated secondary antibody (25 min at room temperature), and the presence of the secondary antibody revealed by streptavidin–horseradish peroxidase conjugate using diaminobenzidine as chromogen (Dako, Glostrup, Denmark), after which they were counterstained with Harris hematoxylin.

### Preparation of human brain homogenates and biochemical analysis

Parietal cortex samples were individually homogenized at 20% weight/volume (w/v) in a sterile 5% glucose solution (Virbac, Boulogne, France) in a ribolyser sample homogenizer (Hybaid, FastPrep FP120, Bio 101, Thermo Savant). Brain homogenates were then aliquoted into sterile polypropylene tubes and stored at − 80 °C until use.

Brain homogenates were further characterized by biochemistry. For Aβ, brain 20% homogenates were diluted in 6.8 M guanidine and 68 mM TrisHCl to obtain a final concentration of 5 M guanidine, protease inhibitor (Complete Mini, Sigma Aldrich, MO, USA) added, and vortexed for 3 h at room temperature. Aβ immunoquantification was performed in duplicate with human Aβ_1–42_ ELISA kits (Invitrogen, Carlsbad, CA, USA) and Aβ_1–40_ ELISA kits (Invitrogen) according to the manufacturer’s instructions. For tau characterization, brain homogenates were sonicated on ice for 5 min, centrifuged for 5 min at 3000 x g at + 4 °C, diluted in 20 mM Tris/2% SDS and sonicated on ice for 5 min. Samples were diluted to 1 μg/μL, diluted in 2X lithium dodecyl sulfate (LDS, Thermo Fisher Scientific, Villebon sur Yvette, France) buffer with reducers, and heated at + 100 °C for 10 min. Ten μg of samples were loaded on Criterion gels (Biorad, Hercules, CA, USA) and migrated in MOPS buffer for 90 min at 165 V on ice. After protein transfer on nitrocellulose sheets, either pS396 (Life technologies, Carlsbad, CA, USA) or tau-Cter antibodies [23] were incubated overnight at + 4 °C. A peroxidase coupled secondary anti-rabbit antibody (ref-23817-2, Biovalley, Nanterre, France) was then applied for 45 min at room temperature. Immunoblotting (or western blotting) was revealed by ECL. GAPDH (ref sc-25778, Santacruz, Nanterre, France) was used as a loading control. Operators were blinded to the status of the patients. Brain homogenates were also characterized for the presence of prion proteins by western blotting. Fragments from frontal cortex and cerebellum were homogenized in an isotonic glucose solution. Scrapie-associated fibrils were purified after digestion by proteinase K. Polyacrylamide gel electrophoresis was performed and PrPres was evaluated with 3F4 (Signet, 0.04 μg/ml) antibodies and revealed by electrochemoluminescence. Tau was characterized by western blotting using pS396 (Life technologies, Carlsbad, CA, USA) or tau-Cter antibodies [23]. Brain homogenates were also characterized for the presence of Prion proteins by western blotting according to protocols routinely used in the GIE NeuroCEB Brain Bank.

### Ethical statement for animal experiments

All animal experiments were conducted in accordance with the European Community Council Directive 2010/63/UE. Animal care was in accordance with institutional guidelines and experimental procedures were approved by local ethical committees (authorizations 12–089; ethics committees CEtEA-CEA DSV IdF N°44, France, and agreement APAFIS#2264–2015101320441671 from CEEA75, Lille, France).

### Animals and overall experimental plan

Experiments were conducted on 12 middle-aged mouse lemurs (age = 3.5 ± 0.2 years; males were used in the study as, in our colony, females are reserved for breeding). They were all born and bred in a laboratory breeding colony (UMR 7179 CNRS/MNHN, France; European Institutions Agreement #962773). Mouse lemurs were maintained at a constant temperature of 24–26 °C and relative humidity of 55% and were housed in individual cages with an enriched environment (jumping and hiding). Seasonal lighting (summer: 14 h of light/10 h of dark; winter: 10 h of light/14 h of dark) was applied to coincide with the seasonal rhythm of the animals. Their diet consisted of fresh apples and a homemade mixture of banana, cereals, eggs, and milk and animals had free access to tap water. Before entering the study, all animals were examined for health and given an ophthalmological examination. None of them were previously involved in pharmacological trials or invasive studies. The experiment was based on the inoculation of human brain homogenates from Alzheimer’s disease patients (AD-inoculated group) or a control subject (CTRL-inoculated group) into the brains of mouse lemurs (*n* = 6 animals per group). Group assignments of the animals were performed to obtain two homogeneous groups based on pre-inoculation learning abilities. Longitudinal behavioral, EEG, and morphological MRI studies were performed up to 18 mpi, followed by immunohistopathological examinations of brain tissues (age at death = 5.0 ± 0.2 years, Additional file 1: Table S2), with investigators blind to the group assignment when assessing these outcomes. Five year-old mouse lemurs are considered middle-aged and they usually do not display cerebral atrophy, β-amyloid or tau lesions. We chose to inoculate young adults (3.5 ± 0.2 years) and to follow them during 18 months when they reached a middle-aged stage (age at euthanasia around 5 years) in order to avoid any drawback linked to aging that could affect cerebral atrophy and/or neuropathological status. Two control-inoculated animals were euthanized for ethical reasons at 12 mpi due to an abdominal infection following self-removal of abdominal sutures after wireless telemetry transmitter explantation. These animals were thus not evaluated by MRI at 12, 15, or 18 mpi or for behavioral studies at 18 mpi.

Mouse experiments were performed in eight-week-old APP/PS1_dE9_ mouse model of β-amyloidosis (*n* = 21) [24] and five-week-old Tau30^+/+^ mouse model of tauopathy (*n* = 15) [25]. The same brain homogenates as those used in lemurs were inoculated in the mice. APP/PS1_dE9_ mice were followed-up for 4 months, while Tau30^+/+^ mice were followed-up for 1 month before immunohistopathological examinations of their brains.

### Stereotaxic injections in mouse lemurs

In mouse lemurs, brain homogenates were injected using stereotaxic surgery in four different sites surrounding the parietal cortex in order to spread the homogenates in wide brain regions. The 20% aliquoted homogenates were diluted to 10% (w/v) in sterile Dulbecco’s phosphate-buffered saline (PBS, Gibco, ThermoFisher Scientific, France) extemporaneously. Six animals received brain extract from the control patient (CTRL-inoculated group) and six received brain extract from Alzheimer’s disease patients (*n* = 3 per patient, AD-inoculated group). Animals were fasted the day before surgery. Pre-anesthesia (atropine, 0.025 mg/kg, subcutaneous injection) was performed 30 min before anesthesia (Isoflurane, Vetflurane, 4.5% for induction and 1–2% for maintenance) as described previously [26]. Animals were then placed in a stereotaxic frame (Phymep, France). Burr holes were drilled 1.25 mm in front of the interaural axe. Using 26-gauge needles, 6.5 μL of 10% w/v brain homogenates were injected bilaterally (L +/− 2.5 mm) 3 mm below the brain surface. Homogenates were inoculated at 1 μL/min. Needles were kept in place for additional 2 min before they were slowly moved 2 mm above where bilateral injections were also performed (same volume and injection speed as described above). Needles were kept in place for additional 5 min before being slowly removed. Respiration rate was monitored during the entire procedure and body temperature was maintained at 37 ± 0.5 °C with a heating blanket or air-heating system. The surgical area was cleaned before and after surgery (iodinate povidone, Vetedine, Vetoquinol, France), the incision sutured, and the animals were placed in an incubator at 25 °C and monitored until recovery from anesthesia. Mouse lemurs were followed up to 18 months after inoculation (Additional file 1: Table S2).

### Stereotaxic injections in mice

Control- or Alzheimer’s disease-brain homogenates (prepared identically to those injected into the mouse lemurs) were injected bilaterally in the dorsal hippocampus (AP − 2 mm, DV − 2 mm, L +/− 1 mm [27]) of eight-week-old female APP/PS1_dE9_ mice (*n* = 21) and five-week-old Tau30^+/+^ mice (*n* = 15) of both sexes. Mice were randomly assigned to control- (APP/PS1_∆E9:_ *n* = 6 and Tau30^+/+^: *n* = 5) or Alzheimer’s disease-inoculated groups (APP/PS1_∆E9_: *n* = 6 and 9 per patient, Tau30^+/+^: *n* = 5 per patient). They were anaesthetized by intraperitoneal ketamine-xylazine injection (Imalgène 1000, Merial, France (1 mg/10 g); 2% Rompun, Bayer Healthcare, Leverkusen, Germany (0.1 mg/10 g)) and placed in a stereotaxic frame (Phymep, France). Respiration rate was monitored and body temperature was maintained at 37 ± 0.5 °C with a heating blanket during surgery. After making a midline incision of the scalp, burr holes were drilled in the appropriate location. Bilateral intrahippocampal injections of 2 μL 10% brain homogenates were performed with a 26-gauge needle. The surgical area was cleaned before and after surgery (iodinate povidone, Vetedine, Vetoquinol, France), the incision sutured, and the animals placed in an incubator (temperature 25 °C) and monitored until recovery from anesthesia.

### Behavioral evaluations

#### Accelerating rotarod task

Mouse lemurs were evaluated with the accelerating rotarod task (model 7750, Ugo Basile, Italy) before inoculation and every 6 mpi. Animals were placed on a 5-cm-diameter rotating cylinder turning at 20 rotations per minute (rpm). The rod then accelerated steadily up to 40 rpm until the end of the test, which was reached when the animal fell or gripped onto the rod during at least three consecutive turns without stabilizing its balance. Latency to fall off or grip the rod was recorded for each trial. Animals underwent five consecutive trials and the best result was recorded with values expressed in seconds. The apparatus was cleaned with ethanol between each trial and each animal.

#### Visual discrimination test

The cognition of mouse lemurs was evaluated in an apparatus (Additional file 2: Figure S1a) adapted from the Lashley jumping stand apparatus [28], which is a vertical cage made of plywood walls, except for the front panel, which is a one-way mirror allowing observation. Two discrimination tasks were performed: a learning task and a long-term memory task. These tests involved a succession of visual discrimination tasks during which the mouse lemur had to jump from a heightened central platform to one of two lateral boards, one of which allowed access to a reinforcing chamber containing a positive reward (the possibility of reaching a safe nestbox for a 2-min rest). As mouse lemurs prefer confined spaces, reaching a nest when placed in an open space is a strong motivator for behavioral testing. If no jump is performed within 1 min, the central station can be progressively and gently tilted downwards creating a slippery slope, encouraging the mouse lemur to jump. Boards can be covered with removable and easily-discriminable patches of varied shape, texture, and pattern (i.e. visual discrimination clues). Each board can be locked in a stable position or unlocked to become unstable and fall if a lemur jumps on it. For a pair of patches, one is always associated with the stable board, giving access to the nest (positive result), and the other with the unstable board that falls when the lemur jumps on it (negative result). During a discrimination task, the mouse lemur had to identify the positive stimulus which signaled access to the nest. Left/right locations of the stimuli were randomly alternated during the attempts with the restriction of no more than three consecutive trials in the same configuration. Testing continued until a success criterion - defined as eight correct choices out of 10 successive attempts - was achieved. Before the first test, lemurs underwent a habituation session composed of seven trials. For the first four trials, only one fixed central board was attached just below the nestbox opening. In trial 1, a cylindrical rod connected the central station to the board so that no jump was required to reach the nestbox. In trial 2, the rod was removed so that the mouse lemur had to jump onto the central board to access the nestbox. In trials 3 and 4, an opaque vertical screen was added above the middle of the board masking the nestbox opening. The mouse lemur had to jump onto the board and then walk under the screen to access the nestbox. For the final three trials, the fixed landing platform was placed alternately to the left or right of the nestbox opening which was masked by the opaque screen. After the habituation session, mouse lemurs underwent the first discrimination learning task – distinguishing between a pair of patches – to test their learning abilities. This task was performed before inoculation and then at 6, 12, and 18 mpi with a new set of discrimination task stimuli each time (i.e. a new pair of patches). Long-time retention was also evaluated at the three post-inoculation time points through recall of the discrimination task from 6 months prior (Additional file 2: Figure S1b).

#### Electroencephalography (EEG)

EEG studies were conducted in mouse lemurs using telemetric devices as described before [29, 30]. Animals received pre-anesthesia (5 mg/mL Diazepam, Roche, France, intramuscular injection of 200 μL/100 g) and were then anesthetized with isoflurane. A wireless telemetry transmitter (2.5 g, PhysioTel F20-EET, Data Science, St Paul, MN, USA), equipped with simultaneous recording for one EEG and one electromyogram (EMG) channel (1–500 Hz sampling rate), was implanted in the abdominal cavity. The electrode leads were threaded subcutaneously from the abdomen to the skull. Electrodes were placed on the dura mater of the anterior frontal cortex according to a stereotaxic atlas of the mouse lemur brain and secured using dental cement [31]. The frontal cortex, and not the parietal cortex in which brains homogenates were inoculated, was chosen for this evaluation to focus on the impact of the brain homogenate inoculation on cerebral networks, including those distant from the inoculation site. For EMG recording, bipolar electrodes were sutured into the neck muscles using non-absorbable polyamide sutures. Animals were monitored for respiration rate and body temperature during surgery, observed until anesthesia recovery, and allowed to recover from surgery for 1 week before recording. EEG and EMG data were continuously collected using PC running Dataquest software (Data Science International, St Paul, MN, USA) linked to a receiver base (RPC-1, Data Science, St Paul, MN, USA), placed on the floor of the home cage inhabited by the implanted animals. Electrodes and the telemetry transmitter were removed after 1 week of recording under the same surgical conditions as for implantation. The EEG data were analyzed with Neuroscore v2.1.0 (Data Science International, St Paul, MN, USA). Analysis focused on the active state, determined by locomotor activity recording (included in the telemetry data of EMG recordings). EEGs were performed before inoculation and 6 and 12 mpi. We focused on delta (0.5–4 Hz), theta (4–8 Hz), alpha (8–12 Hz), sigma (12–16 Hz), and beta (16–24 Hz) frequency waves. At each time point, each wave was normalized according to mean values of the control-inoculated animals. The operator was blinded to the group attribution during EEG signal processing.

#### Morphological MRI

Brain images were recorded on a 7.0 Tesla spectrometer (Agilent, USA) using a four-channel phase surface coil (RapidBiomedical, Rimpar, Germany) actively decoupled from the transmitting birdcage probe (RapidBiomedical, Rimpar, Germany). Two-dimensional fast spin-echo images were recorded with an isotropic nominal resolution of 230 μm (128 slices, TR/TE = 10000/17.4 ms, rare factor = 4; field of view = 29.4 × 29.4 mm^2^, matrix = 128 × 128, slice thickness = 230 μm, acquisition time = 32 min). MR images were zero-filled to reach an apparent isotropic resolution of 115 μm. Animals were anesthetized and monitored as described for stereotaxic injections. MR images were recorded for each animal before inoculation, 15 days after inoculation and then every 3 months until 18 mpi.

Images were analyzed using voxel-based morphometry by applying SPM8 (Wellcome Trust Institute of Neurology, University College London, UK, www.fil.ion.ucl.ac.uk/spm) with the SPMMouse toolbox (http://spmmouse.org) for animal brain morphometry [32]. Fifteen-day post-inoculation images were not included in this analysis, as they were only used to ensure accurate injection cannula placement and the lack of acute lesions following surgery.

The brain images were segmented into gray (GM) and white matter (WM) tissue probability maps using locally developed priors, then spatially transformed to the standard space, defined by Sawiak et al., using a GM mouse-lemur template [32]. Affine regularization was set for an average-sized template, with a bias non-uniformity FWHM cut off of 10 mm and a 5 mm basis-function cut off and sampling distance of 0.3 mm. The resulting GM and WM portions were output in rigid template space, and DARTEL [33] was used to create non-linearly registered maps for each subject and common templates for the cohort of animals. The warped GM portions for each subject were adjusted using the Jacobian determinant from the DARTEL registration fields to preserve tissue amounts (“optimized VBM” [34]) and smoothed with a Gaussian kernel of 600 μm to produce maps for analysis.

A general linear model was designed to evaluate relative changes in GM values as a function of time between the control- and Alzheimer’s disease-inoculated groups. Longitudinal follow-up of each animal was considered in the design matrix, and total intracranial volumes were treated as covariates of no interest. This type of analysis produces t-statistic and color-coded maps that are the product of a statistical analysis performed at every voxel in the brain. Contiguous groups of voxels that attain statistical significance, called clusters, are displayed on brain images.

With the general linear model, if the brain of one animal is defined by the number “j”, and the location of a pixel is defined as “k”. The signal within a pixel (${\mathrm{Y}}_j^k\Big)$ can be explained by the following equation

$$ {\mathrm{Y}}_j^k={x}_{j,1}{\beta}_1^k+{x}_{j,2}{\beta}_2^k+{T}_j^1{\beta}_3^k+{T}_j^2{\beta}_4^k+\dots +{T}_j^6{\beta}_8^k+{T}_j^7{\beta}_9^k+\dots +{T}_j^{12}{\beta}_{14}^k+{TIV}_j{\beta}_{15}^k+{\epsilon}_j^k $$

With ${\beta}_1^k$ = Alzheimer’s disease brain inoculation effect (*n* = 42 images); ${\beta}_2^k$ = control brain inoculation effect (*n* = 36 images); ${\beta}_3^k$ = Longitudinal follow-up for Alzheimer’s disease-brain inoculated animal #1 (*n* = 7 values, i.e. at 0, 3, 6, 9, 12, 15, and 18 mpi); ${\beta}_4^k$ = Longitudinal follow-up for Alzheimer’s disease-brain inoculated animal #2 (*n* = 7 values, i.e. at 0, 3, 6, 9, 12, 15, and 18 mpi);...; ${\beta}_8^k$ = Longitudinal follow-up for Alzheimer’s disease-brain inoculated animal #6; ${\beta}_9^k$ = Longitudinal follow-up for control animal #1;…; ${\beta}_{14}^k$ = Longitudinal follow-up for control animal #6; …; ${\beta}_{15}^k$ = total intracranial volume (TIV) for each animal. The Alzheimer’s disease-brain inoculation effect is defined by ${x}_{j,1}{\beta}_1^k$ and the control-brain inoculation effect is defined by ${x}_{j,2}{\beta}_2^k$. Thus, if the image corresponds to an Alzheimer’s disease-inoculated animal, *x*_*j*, 1_ = 1 and *x*_*j*, 2_ = 0, if the image corresponds to a control-inoculated animal, *x*_*j*, 1_ = 0 and *x*_*j*, 2_ = 1. ${T}_j^x$ is the time post-inoculation for each animal x. Otherwise, ${T}_j^x=0$. TIV corresponds to the total intracranial volume value for each animal. It was similar for the different images from the same animal followed-up longitudinally. The term ${\epsilon}_j^k$ corresponds to the “error” of the measure for each animal.

As an example, on the basis of this model, for image 1 of animal 1 (Alzheimer’s disease group, image before inoculation (${T}_1^1=1$)): ${\mathrm{Y}}_1^k={\beta}_1^k+1\ {\beta}_3^k+{TIV}_1{\beta}_{15}^k+{\epsilon}_1^k$; for image 2 of animal 1 (Alzheimer’s disease group, image at 3 mpi (${T}_2^1=3\  months=92$ days)): ${\mathrm{Y}}_2^k={\beta}_1^k+92\ {\beta}_3^k+{TIV}_2{\beta}_{15}^k+{\epsilon}_2^k$; for image 3 of animal 1 (Alzheimer’s disease group, image at 6 mpi (${T}_3^1=6\  months=183$ days)): ${\mathrm{Y}}_3^k={\beta}_1^k+183\ {\beta}_3^k+{TIV}_3{\beta}_{15}^k+{\epsilon}_3^k$ … for image 1 from animal 7 (control group image before inoculation (${T}_{43}^7=1$)):): ${\mathrm{Y}}_{43}^k={\beta}_2^k+1\ {\beta}_9^k+{TIV}_{43}{\beta}_{15}^k+{\epsilon}_{43}^k$; for image 2 of animal 7 (control group image at 3 mpi (${T}_{44}^7=3\  months=92$ days)): ${\mathrm{Y}}_{44}^k={\beta}_2^k+92\ {\beta}_9^k+{TIV}_{44}{\beta}_{15}^k+{\epsilon}_{44}^k$.

A contrast defines a linear combination of *β* as *c*^*T*^*β*. For example, the test evaluating whether the probability of pixels from animals inoculated with Alzheimer’s disease-brains to be GM is lower than that for control-brain inoculated animals is defined using a contrast *c*^*T*^*β* = {− 1 1 0...]^T^. The Null hypothesis is *H*_0_ : *c*^*T*^*β* = 0, whereas the alternative hypotheses is *H*_1_ : *c*^*T*^*β* > 0. This hypothesis is tested with:

$$ T=\frac{c^T\beta }{\sqrt{\sigma^2{c}^T{\left({X}^TX\right)}^{-1}c}}=\frac{\mathrm{contrast}}{\sqrt{\mathrm{estimated}\ \mathrm{variance}}} $$

This analysis allows the removal of confounding effects, such as repetition of the measures during longitudinal evaluation of the same animal (i.e. ${\beta}_3^k$, ${\beta}_4^k$,…, ${\beta}_8^k$, ${\beta}_9^k$,…, ${\beta}_{14}^k$) or TIV (${\beta}_{15}^k$) from the raw data. In other words, volumetric scans were entered as the dependent variable. The treatment groups of the animals (Alzheimer’s disease or control-brain inoculation) were the independent variables. Longitudinal follow-up effect and TIV were covariates.

One-tailed t-test contrasts were set up to find areas in which probability values from GM maps were different in Alzheimer’s disease and control-brain inoculated animals (i.e. *c*^*T*^*β* = {−1 1 0...]^T^ or {1–1 0...]^T^ contrasts). To control for multiple comparisons, an adjusted *p*-value was calculated using the voxel-wise false discovery rate (FDR-corrected *p* < 0.05), with extent threshold values of 10 voxels, meaning that clusters required 10 contiguous voxels to be selected as relevant [35]. Voxels with a modulated GM value below 0.2 were not considered for statistical analysis. The operator was blinded to the group attribution during image processing.

The rate of atrophy evolution over time was then further evaluated based on changes of the Jacobian determinant. More specifically, the change in the Jacobian determinant was calculated for each subject relative to baseline and averaged groups, yielding mean volume changes from baseline to time *t* post-inoculation of $\Delta {\overline{J}}^{AD}(t)$ and $\Delta {\overline{J}}^{CTRL}(t)$ for Alzheimer’s disease- and control-inoculated groups, respectively. Subtracting these highlighted voxels showing differential atrophy between groups at each stage. We evaluated differences in atrophy over the first 6 months, further atrophy that occurred from six to 12 months, and later atrophy occurring from 12 to 18 months: i.e. $\Delta {\overline{J}}^{AD}(6)-\Delta {\overline{J}}^{CTRL}(6)$, $\left(\Delta {\overline{J}}^{AD}(12)-\Delta {\overline{J}}^{CTRL}(12)\right)-\left(\Delta {\overline{J}}^{AD}(6)-\Delta {\overline{J}}^{CTRL}(6)\right)$ and $\left(\Delta {\overline{J}}^{AD}(18)-\Delta {\overline{J}}^{CTRL}(18)\right)-\left(\Delta {\overline{J}}^{AD}(12)-\Delta {\overline{J}}^{CTRL}(12)\right)$.

#### Immunohistochemistry and biochemistry

Five mouse lemurs from each group were studied by immunohistochemistry. The last animal from each group was not evaluated by immunohistochemistry as its brain was sampled for future inoculations in new cohorts of animals (second passages). All mice were included in immunohistochemical analyses. Each animal studied by immunohistochemistry was euthanized with an overdose of sodium pentobarbital (100 mg/kg intraperitoneally) followed by intracardiac perfusion with 4% paraformaldehyde in PBS. After overnight post-fixation, brains were cryoprotected using 15 and 30% sucrose solutions. Brain coronal sections (40-μm-thick) were cut on a sliding freezing microtome (SM2400, Leica Microsytem). Twenty series of sections were performed. The floating histological serial sections were preserved in a storage solution (30% glycerol, 30% ethylene glycol, 30% distilled water, and 10% phosphate buffer) at − 20 °C until use.

Serial sections of the entire brains of mouse lemurs were used for Aβ (4G8), tau (AT8, MC1, and AT100), glial fibrillary acidic protein (GFAP), and neuronal nuclei (NeuN) using immunohistochemistry. One series of sections (i.e. one section every 20th sections, which represents approximately 12 sections) was used for each staining, except for NeuN that used 4 series (i.e. one section every 5th sections). Monoclonal antibody AT8 (Thermo Scientific MN1020B, USA) recognizes phosphorylated residues serine 202 and threonine 205 of Tau. The monoclonal antibody AT100 (Thermo Scientific MN1060, USA) recognizes phosphorylated residues threonine 212 and serine 214 of Tau. The monoclonal antibody MC1 was a generous gift from Peter Davies and recognizes conformational changes in residues seven to nine and 313–322. Human sections were used as positive controls. Free-floating sections were rinsed in 0.1 M PBS and incubated in 0.3% hydrogen peroxide for 20 min. For 4G8 staining, sections were pre-treated with 80% formic for 2 min. Pre-treatment with PBS - Triton 0.5% (Triton X-100, Sigma Aldrich, MO, USA) and 3% bovine serum albumin (BSA) blocking was performed at + 4 °C for 30 min before a 2 day-incubation with either biotinylated 4G8 (Covance, NJ, USA, 1/250), GFAP (Dako, Denmark, 1/5000), or NeuN (Abcam, Cambridge, UK, 1/2000) antibodies. Sections stained for tau lesions were pre-treated with 1x Citrate Buffer, in a 100 °C water bath for 30 min for antigen unmasking. Then they were processed with PBS - Triton 0.2% (Triton X-100, Sigma Aldrich, MO, USA) and 10% normal goat serum (NGS) 10% blocking at + 4 °C for 1 h before a 3 day-incubation at 4 °C with AT8 (1/500 in PBS - Triton 0.2% and NGS 5%), MC1 (1/500 in PBS - Triton 0.2% and NGS 5%) or AT100 (1/200 in PBS - Triton 0.2% and NGS 5%). Sections were incubated in biotinylated anti-mouse or anti-rabbit secondary antibodies (IgG, Vector Laboratories, Burlingame, CA, USA) in PBS - Triton 0.2% for 1 h before revelation. The ABC Vectastain kit (Vector Laboratories, Burlingame, CA, USA) was used to amplify DAB revelation (DAB SK4100 kit, Vector Laboratories, Burlingame, CA, USA). Sections stained for Tau were also counterstained with cresyl violet for 45 s or counterstained with Olig2 (Millipore AB9610, USA, 1/500) to detect oligodendrocytes. Images of stained serial sections were digitized with an Axio ScanZ.1 slide scanner (Zeiss, Jena, Germany) at X20 (0.22 μm in plane resolution). The scanned files were exported as jpeg RGB images with a 30% compression (0.73 μm in plane resolution) using Zen 2.0 (Zeiss, Jena, Germany).

Quantification of intracellular APP/Aβ deposits was performed blind using 4G8 stained-sections and ImageJ 1.52b (https://imagej.nih.gov/ij/). Briefly, 4G8-positive objects were segmented using the same threshold for each animal. Then masks were created to exclude brain vessels from parenchyma. Overlap between 4G8-positive objects and these masks was used to evaluate 4G8-positive staining either in brain parenchyma (ImageJ ROI manager and analyze particles function). Three brain regions (caudate nucleus, putamen, and hippocampus) were studied. Quantification of GFAP-stained sections was blindly performed using ImageJ 1.52b. Each structure (frontal cortex, entorhinal cortex, amygdala, hippocampus, posterior cingulate, and retrosplenial cortices) was manually defined for both hemispheres. GFAP staining density was evaluated as relative optical density (log (255/(255-GFAP gray level))) in each structure.

Microglial reaction was further evaluated in mouse lemurs by Western blot analysis. Proteins were extracted from two floating brain sections from each mouse lemur, taken at the level of the inoculation site (Qproteome FFPE – Tissue Extraction Buffer, Qiagen). Extracted total proteins were detected by immunoblotting using SDS–PAGE (Criterion TGX Stain-Free Precast Gel 4–20%, Bio-Rad), UV activation, nitrocellulose membrane transfer (Trans-Blot Turbo RTA Transfer Kit, Bio-Rad) and by blotting with anti-Iba1 rabbit polyclonal antibody (Wako, 1:1000) followed by anti-rabbit secondary antibody (Invitrogen, 1:5000) and by Clarity Western ECL chemiluminescence revelation (Bio-Rad). Proteins were migrated together with a molecular weight marker (Precision Plus Protein Standards Unstained, Bio-Rad) ranging from 10 to 250 kD. Images of the blots were digitized with a ChemiDoc Imaging System (Bio-Rad) and quantified with the Image Lab Software 5.2.1 (Bio-Rad).

Serial sections of the entire brain of APP/PS1_∆E9_ mice were stained for the evaluation of Aβ pathology, as well as inflammation (Iba1 and GFAP). Brain sections were rinsed with PBS, and then incubated in 0.3% hydrogen peroxide for 20 min. Sections were then blocked with PBS-0.5% Triton (Triton X-100, Sigma, St Louis, MO, USA) and 4.5% normal goat serum (NGS) for 30 min before overnight incubation with BAM10 (Sigma, A3981, 1/10.000), Iba1 (Wako, VA, USA, /1000), or GFAP (Dako, Denmark, 1/10000) antibodies at 4 °C. Sections were rinsed with PBS and then successively incubated with 1/1000 anti-mouse IgG secondary antibody (BA-9200; Vector Labs) at room temperature for 1 h and ABC Vectastain (Vector Labs) before DAB revelation (DAB SK4100 kit, Vector Labs). Images of stained sections were digitized with a Zeiss Axio Scan.Z1 (Zeiss, Jena, Germany) whole slide imaging microscope at X20 (0.22 μm in plane resolution). The scanned files were exported as jpeg RGB images with a 30% compression (0.73 μm in plane resolution) using Zen 2.0 (Zeiss, Jena, Germany). Sections stained for Aβ were blindly analyzed and Aβ in the hippocampus quantified using ImageJ software [36]. Segmentation of β-amyloid deposits was performed in two sections corresponding to AP -1.70/− 2.30 mm [27]. It was based on the determination of a threshold defined as T = M_signal_ + 10xSD_signal_, where M_signal_ and SD_signal_ represent the mean and standard deviation of the signal within a CA1 region of interest in which β-amyloid deposits were not visible.

Neuroinflammation was blindly evaluated after manual adjustment of a threshold adjusted to select the stained microglial and astroglial cells. Their load was quantified by densitometry in the inoculated brain region (CA1) using Explora Nova Mercator.

Brains sections from tau30^+/+^ mice were stained with anti-AT8 antibodies. Serial sections from the entire brain were washed in PBS-0.2% Triton and treated for 30 min with H_2_O_2_ (0.3%). Non-specific binding was then blocked using the MOM kit (Vector MKB2213) (1/100 in PBS, Vector) for 60 min. Incubation with AT8 (Thermo Scientific MN1020, 1/500) in PBS-0.2% Triton was performed overnight at 4 °C. After several washes, labelling was amplified by incubation with an anti-mouse biotinylated IgG (1/400 in PBS-0.2% Triton, Vector) for 60 min followed by the application of the ABC kit (1:400 in PBS, Vector) prior to visualization with 0.5 mg/ml DAB (Vector) in 50 mmol/L Tris-HCl, pH 7.6, containing 0.075% H_2_O_2_. Brain sections were mounted onto gelatin-coated slides, stained for 1 min in a cresyl violet solution (0.5%), washed in water containing 2% acetic acid, dehydrated by passage through a graded series of alcohol and toluene solutions and mounted with Vectamount (Vector) for microscopic analysis. Images were digitized with a Zeiss Axio Scan.Z1 (Zeiss, Jena, Germany) whole slide imaging microscope at X20 (0.22 μm in plane resolution). The density of AT8-positive cell-soma profiles in the hippocampus was evaluated in two sections corresponding to AP -1.70/− 2.30 mm [27]. AT8-positive cell soma were manually counted (“PointPicker” tool from ImageJ [36]) and expressed as the total number of AT8-positive cell soma profiles visible in the dorsal hippocampus.

#### Stereological counting of NeuN-positive neurons

The optical fractionator method [37, 38] was used to obtain an unbiased stereological estimate of the total number of NeuN-positive cells in the CA1/2 and CA3 layers of the hippocampus, layers I, II, and III-VI of the entorhinal cortex, and the cingulate/retrosplenial cortex. Measurements were performed on the left hemisphere of the brain. Cells were counted using a Leica DM6000 microscope equipped with a digital color camera (MicroFireTM, Optronics, Goleta, CA, USA), an x-y motorized stage controller, a motorized z-axis, and Mercator stereology software (Explora Nova, La Rochelle, France). The regions were delineated using a 4X objective in accordance with the mouse lemur brain atlas [31]. Section thickness (from 11 to 13 μm) was measured at three locations for each section analyzed. Sampling was performed unilaterally within the delineated areas with a 40X oil-immersion objective. Counting frames were adapted for each brain region, and areas of the counting frames (a/f) were 30 × 30 μm^2^ to 100 × 100 μm^2^, depending on the brain region, while sampling areas were separated by x-y steps of 50–50 μm to 150–150 μm, depending on the brain region. The fraction of the section plane sampled (ASF) was calculated as the ratio (a/f)/(x*y) (Additional file 1: Table S3). Disector height was 10 μm with a guard zone of 1 μm from the surface of the section generating counts of 90–2500 sampled cells per animal, depending on the structure, and the mean coefficient of error (CE) of the estimates was 0.06 (Additional file 1: Table S3). The total number of NeuN-positive cells within each region was calculated according to the following formula: Ntot = ΣQ^−^ × 1/SSF × 1/ASF × 1/TSF, in which ΣQ^−^ is the number of sampled cells and SSF is the section sampling fraction. One out of ten sections was sampled, leaving 200 μm intervals between two sampled sections. ASF is the area of the sampling fraction and TSF the thickness of the sampling fraction. Values of SSF, ASF, and TSF for each region are given in Additional file 1: Table S3. All histological data (surface, volume measurements, and cell counts) were performed by an investigator (SL) blind to the group assignment of the animals.

### Statistical analysis

Statistical analyses were performed using GraphPad Prism software (San Diego, CA, USA). In most graphics, data are shown as scatter plots with median and interquartile interval. Control-inoculated animals, as well as animals inoculated with each Alzheimer brain homogenates are displayed with different color codes. Behavioral studies are not displayed as scatter plots but as mean ± standard deviation (SD) to represent cognition evolution over time. Cognitive and motor experiments were evaluated by two-way repeated measures ANOVA (post-inoculation delay, group) followed by Bonferroni’s multiple comparisons post-hoc tests. Data normality and variance homogeneity were evaluated using Shapiro-Wilk and Cochran C tests, respectively, and data from behavioral experiments were reciprocally transformed to obtain normality and variance homogeneity. The values within control-inoculated animals were highly homogeneous for each post-inoculation time. We thus replaced missing data for 18 mpi from the two control-inoculated mouse lemurs that died with the worst values in the control-inoculated group at 18 mpi. EEG, neuronal counts and intracellular β-amyloid load in mouse lemurs, and β-amyloid, tau and inflammation in mice were evaluated by Mann-Whitney tests. Spearman’s rank correlations were performed to examine relationships between EEG and behavioral data. The proportion of mouse lemurs with β-amyloid lesions in the control- and Alzheimer-inoculated groups was compared using the Chi-square test. No statistical methods were used to predetermine sample size. Sample size to compare control and Alzheimer-inoculated mice and mouse lemurs in future studies was estimated on the basis of the experimental results obtained during the current experiments assuming a significance level of 5%, a power of 80% and two-sided tests. Estimations for quantitative values used a two-sample t-test on the basis of the mean and standard deviation obtained for the different measures in this study (BiostaTGV module, https://biostatgv.sentiweb.fr/?module=etudes/sujets#). The standard deviation used for this estimation was the square root of the pooled variance from each group. Estimations for proportional values (proportion of lemurs with Aβ, CAA or Tau in the brain) were based on a chi-squared test (BiostaTGV module based on epiR package 0.9–9.6, https://biostatgv.sentiweb.fr/?module=etudes/sujets#).

## Results

### Characterization of human brain samples and homogenates

Frozen brain tissue samples (parietal cortex) from two Alzheimer’s disease patients (Braak and Braak stage VI, Thal stages 5 and 4, respectively) and one control individual (Braak and Braak/Thal stages 0) were used in the current study. The brains of the Alzheimer’s disease patients displayed typical lesions (β-amyloid plaques and tau tangles) while no lesions were found in the control subject (Additional file 3: Figure S2a-h). One Alzheimer case (AD1) displayed β-amyloid angiopathy in addition to β-amyloid plaques while the second Alzheimer patient (AD2) did not display angiopathy (Additional file 3: Figure S2a-d). Immunohistochemical staining for alpha-synuclein was negative for all brain samples. Biochemical analysis revealed high Aβ_1–42_ and Aβ_1–40_ levels only in Alzheimer’s disease-brain homogenates (Additional file 3: Figure S2i-j). AD1 displayed higher Aβ_1–40_ and lower Aβ_1–42_ levels than AD2 (Additional file 3: Figure S2i-j). Western blotting showed a typical shift in the molecular weight of the Alzheimer tau-Cter triplet [23] and the presence of pathological pS396 tau only in Alzheimer samples (Additional file 3: Figure S2k-l). All brain samples were negative for presence of Prion proteins assessed by western blotting (Additional file 4: Figure S3).

### Alzheimer’s disease brain inoculation effectively induces β-amyloid and tau lesions in transgenic mice

Prior to being used in mouse lemurs, the Alzheimer’s disease and control brains were inoculated in the hippocampus of APP/PS1_dE9_ and Tau30^+/+^ mouse models of β-amyloid and tau lesions. Alzheimer’s disease brain homogenates led to increased Aβ and tau deposition in APP/PS1_dE9_ and Tau30^+/+^ mice (*p* = 0.009 and *p* = 0.0007, respectively, Additional file 5: Figure S4a-f). Within the Alzheimer group, the β-amyloid load was higher in the mice inoculated with the brain presenting with β-amyloid angiopathy as compared to the mice inoculated with the brain without angiopathy (*p* = 0.01). No difference was detected for tau lesions. This experiment confirmed the ability of the Alzheimer’s disease brain homogenates to induce transmission of Alzheimer’s disease-related lesions in mice.

### Alzheimer’s disease brain inoculation induces cognitive alterations in mouse lemurs

**Fig. 1:**
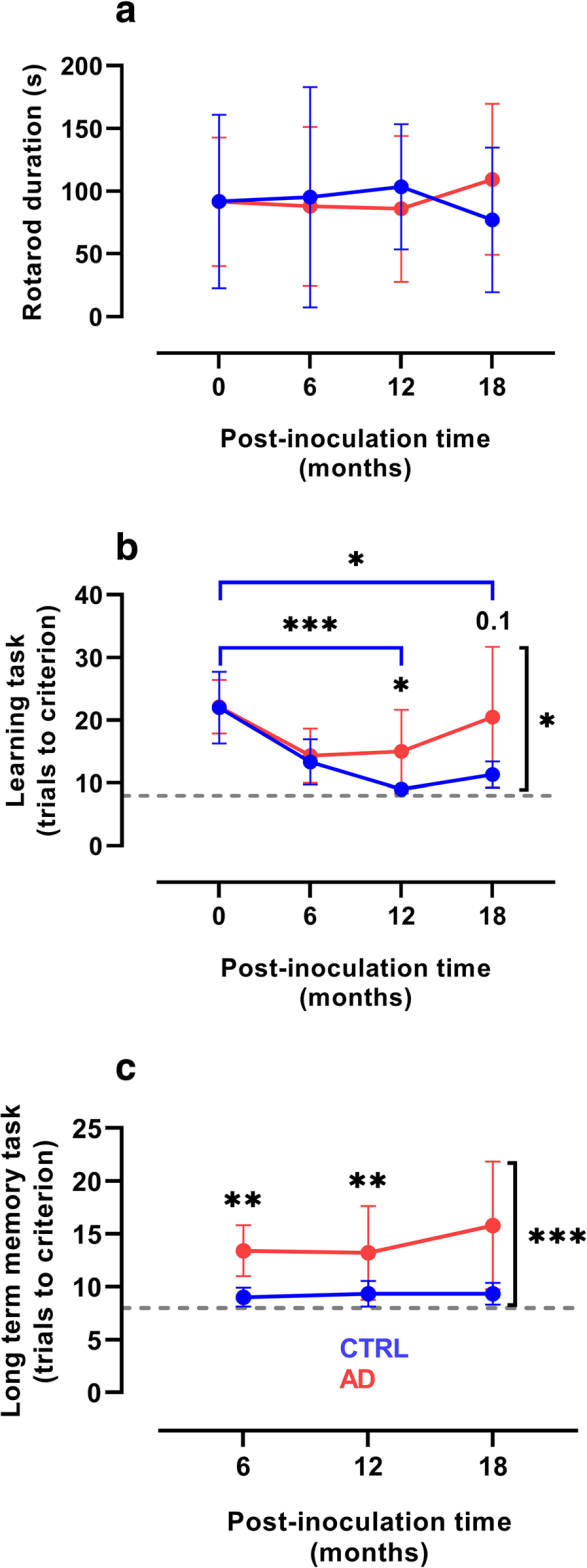
Cognitive dysfunction in Alzheimer-inoculated mouse lemurs. **a** The rotarod test did not reveal any motor dysfunction in either Alzheimer- (AD) or control- (CTRL) inoculated group. **b** Progressive learning impairment in the animals of the Alzheimer-inoculated group. Animals allocated to Alzheimer- and control- inoculated groups performed similarly before inoculation and improved similarly up to 6 mpi. Only the control-inoculated group continued to improve at 12 and 18 mpi (*p* < 0.0001 and *p* = 0.01, respectively). Learning abilities were also lower in the Alzheimer-inoculated group than in the control-inoculated group at 12 mpi (*p* = 0.03) and tended to be lower at 18 mpi (*p* = 0.10). The overall performance of the Alzheimer-inoculated group was significantly worse than that of the control-inoculated group (*p* = 0.02). **c** The overall performance of the Alzheimer-inoculated group in the long-term memory task at 6 and 18 mpi was significantly worse than that of control animals (*p* = 0.0002, 0.0036, and 0.0024, respectively). *p < 0.05; **p < 0.01; ***p < 0.001 (*n* = 6 per group, two-way repeated measures ANOVA with Bonferroni’s post-hoc tests). Plots presents mean ± standard deviation. Dashed lines in (**b, c**) indicate the best possible scores

Following the mouse studies, 12 adult mouse lemurs were inoculated with the same Alzheimer’s disease and control human brain homogenates. Motor functions, assessed with a rotarod test were similar in both groups (Fig. 1a). Cognitive evaluation was performed every 6 months in a jumping-stand apparatus [28] (Additional file 2: Figure S1a) and consisted of two tasks (Additional file 2: Figure S1b). The first was a learning task that rated visual discrimination acquisition abilities, whereas the second was a long-term memory task that assessed retention of the discrimination problem learned 6 months before. Before brain homogenate inoculation, animals assigned to Alzheimer- and control-inoculated groups performed similarly in the learning task (Fig. 1b). Animals from both groups showed similar improvement in their learning abilities 6 months after inoculation (Fig. 1b). However, performance then diverged with control-inoculated animals further improving at 12 mpi, until reaching the best possible scores (thus demonstrating learning set acquisition), whereas the Alzheimer-inoculated group progressively worsened at both 12 and 18 mpi, with overall performance significantly lower than that of control-inoculated group (Fig. 1c). The overall performance of the Alzheimer-inoculated group in the long-term memory task and their performance at 6 and 18 mpi were significantly worse than those of control-inoculated group (Fig. 1c). We did not detect any difference between the scores obtained by the animals inoculated with tissue homogenates from the two Alzheimer brains.

### Alzheimer’s disease brain inoculation alter EEG measures

**Fig. 2:**
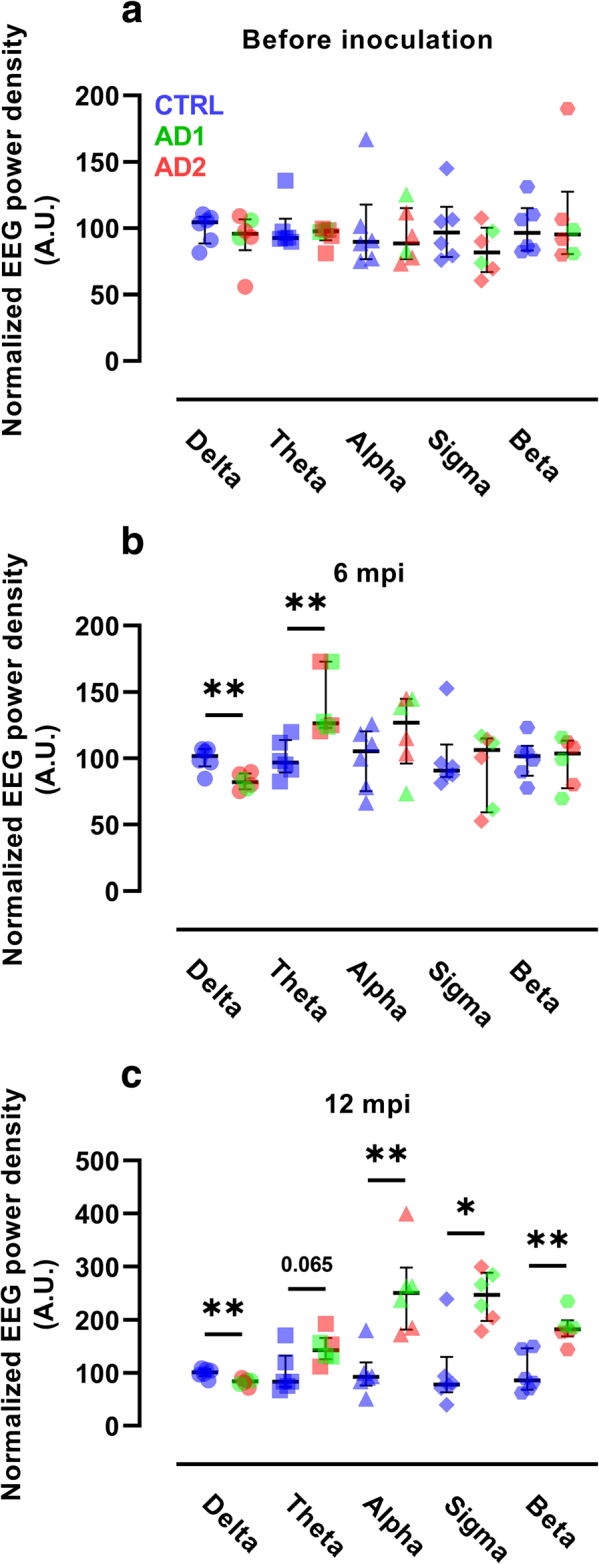
Neuronal activity alterations in Alzheimer-inoculated mouse lemurs. Evolution of EEG frequency power densities in the Alzheimer- (AD) and control- (CTRL) inoculated groups before inoculation (**a**), and 6 (**b**) and 12 (**c**) mpi (*n* = 6 per group). **a** EEG frequency power densities were similar in the two groups before inoculation. **b** At 6 mpi, the Alzheimer-inoculated group showed a lower delta frequency (0.5–4 Hz) and a higher theta frequency (4–8 Hz) than the control-inoculated group (*p* = 0.009 and *p* = 0.002, respectively, Mann-Whitney tests). **c** At 12 mpi, the alterations in delta frequency were maintained (*p* = 0.009, Mann-Whitney test). In addition, alpha (8–12 Hz), sigma (12–16 Hz), and beta (16–24 Hz) frequencies were higher in the Alzheimer-inoculated group than in the control-inoculated group (*p* = 0.004, *p* = 0.015, and *p* = 0.009, respectively, Mann-Whitney tests). *p < 0.05; **p < 0.01. Scatter plots display median and interquartile interval. CTRL-inoculated animals are in blue, AD1-inoculated in green and AD2-inoculated in red

Longitudinal EEG studies were performed in mouse lemurs in order to further evaluate neuronal activity in the Alzheimer- and control-inoculated groups. EEG measures were recorded in the frontal cortex during the active state before the inoculation (Fig. 2a). At 6 mpi, slow wave EEG frequencies were altered, with a lower delta frequency and a higher theta frequency in the Alzheimer- than in the control-inoculated group (Fig. 2b). In addition, the decrease in delta frequency correlated significantly with impairment of long-term memory (*p* = 0.009, Additional file 6: Figure S5). Lemurs from the Alzheimer-inoculated group still displayed a significantly lower delta frequency and a significantly higher fast wave (alpha, sigma, and beta) frequency (Fig. 2c) at 12 mpi than lemurs from the control-inoculated group. We did not detect any difference between the scores obtained by the animals inoculated with tissue homogenates from the two Alzheimer brains.

### Alzheimer’s disease brain inoculation induces a progressive cerebral atrophy

**Fig. 3:**
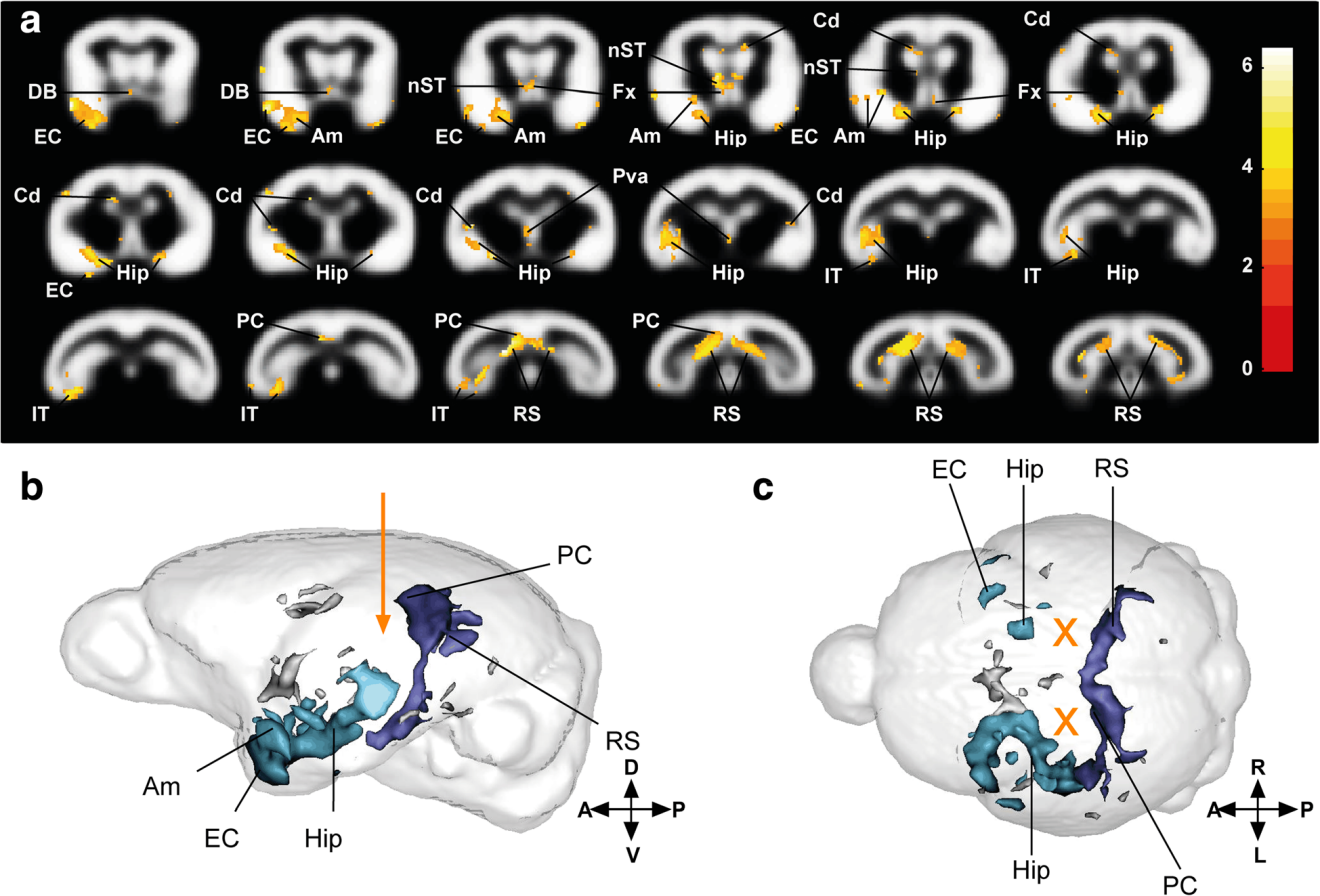
Cerebral atrophy in Alzheimer-inoculated compared to control-inoculated mouse lemurs. **a** Statistical parametric maps depicting regions in which cerebral tissue volume decreased in the Alzheimer-inoculated group compared to the control group. The color-coded blobs show statistical differences between Alzheimer-inoculated animals and control-inoculated mouse lemurs. Images follow the radiological convention (i.e. left hemisphere is on the left). Slices are spaced 0.5 mm apart along the rostro-caudal axis (voxel-based morphometric parameters: FDR-corrected *p* < 0.05; extent threshold k = 10; map represents t values). Lateral **b** and (**c**) dorsal 3D representations of atrophied areas. Orientation of the brain is explained by the crossing arrows, and letters. A: anterior; P: posterior; D: dorsal; V: ventral; L: left; R: right; EC: entorhinal cortex; DB: diagonal band of Broca; Am: amygdala; Hip: hippocampus; nST: nucleus stria terminalis; Fx: fornix; Cd: caudate nucleus; Pva: peri-third ventricle area; IT: inferotemporal cortex; PC: posterior cingulate cortex; RS: retrosplenial cortex. Orange arrow and crosses represent the injection coordinates. Dark blue clusters represent voxels with significant atrophy in the posterior cingulate and retrosplenial cortex. Light blue clusters represent voxels with significant tissue atrophy in the temporal areas of the brain (including the hippocampus, entorhinal cortex, amygdala, and lateral and inferior temporal cortices) as well as the diagonal band of Broca, fornix, and nucleus stria terminalis. Gray clusters represent other significant voxels. Maps derived from MRI recorded on *n* = 6 animals per group at 0, 3, 6, and 9 mpi and *n* = 6 and 4 animals in the Alzheimer- and control-inoculated groups, respectively, at 12, 15, and 18 mpi

Alzheimer’s disease is morphologically characterized by progressive cerebral atrophy affecting the hippocampus and the cortex. We recorded MR images of the brains of lemurs from Alzheimer- and control-inoculated groups every 3 months. Automated voxel-based analysis was performed to evaluate cerebral atrophy. The Alzheimer-inoculated group displayed strong bilateral atrophy of the retrosplenial and posterior cingulate cortices (two areas close to the inoculation sites) relative to the control-inoculated group (Fig. 3a-c, dark blue clusters in b-c; Additional file 1: Table S4). Atrophy also involved temporal regions, including the hippocampus, entorhinal cortex, amygdala, and inferior temporal cortex (Fig. 3a-c, light blue clusters in b-c) with greater atrophy in the left hemisphere as compared to the right hemisphere. We also detected atrophy in the diagonal band of Broca, fornix, stria terminalis, parietal cortex, and caudate nucleus (Fig. 3a-c, gray clusters in b-c; Additional file 1: Table S4). Follow-up of the atrophied regions showed that the process was mild at 6 mpi and mainly developed from 6 to 12 mpi. Thus, atrophy was not associated with an acute effect of the inoculation and continued to spread from 12 to 18 mpi, although to a lesser extent (Additional file 7: Figure S6).

### Alzheimer’s disease brain inoculation induces neuronal loss

**Fig. 4:**
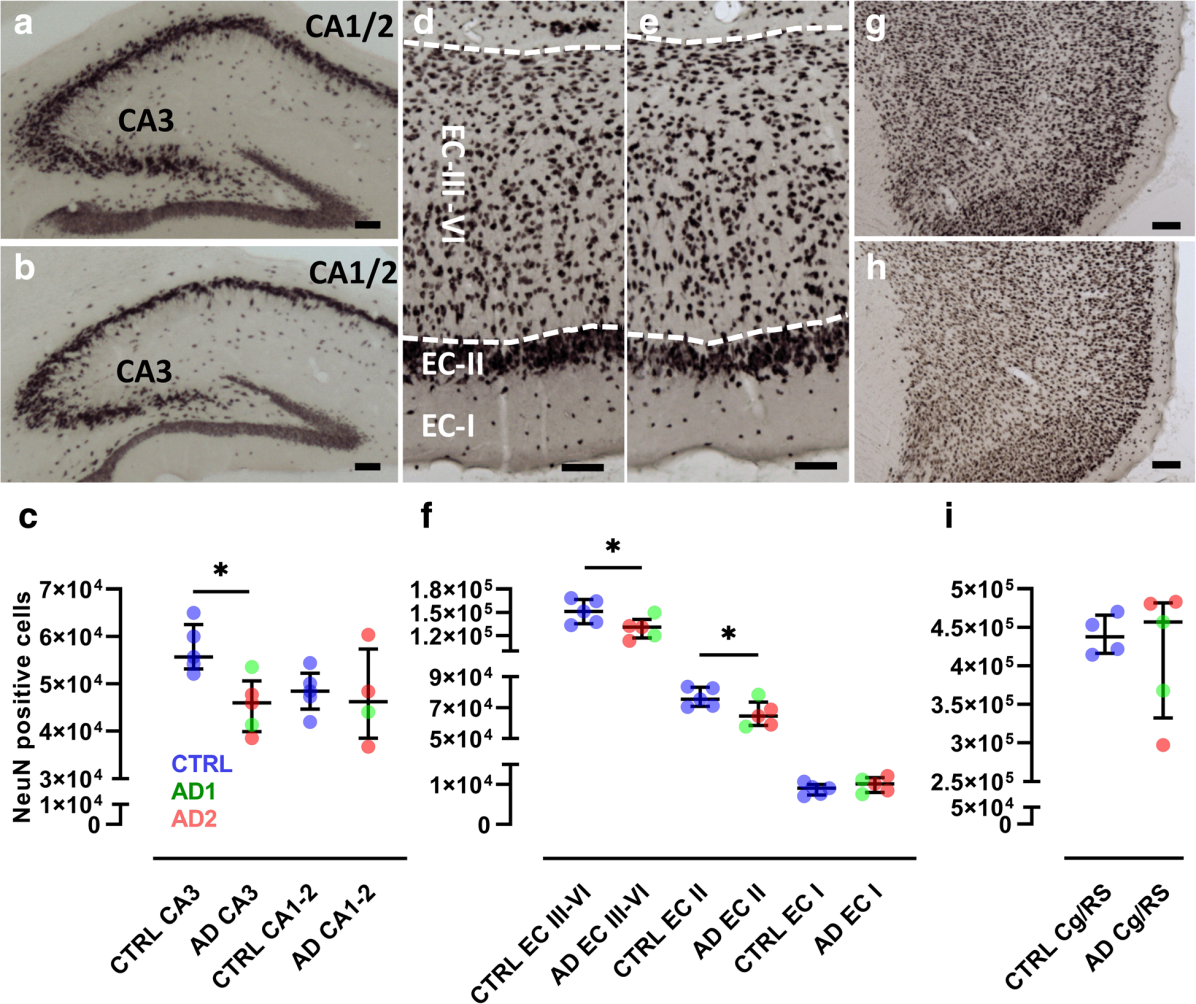
Neuronal loss in Alzheimer-inoculated mouse lemurs. NeuN staining of the dorsal hippocampus in animals of the (**a**) control- (CTRL) and (**b**) Alzheimer-inoculated (AD) groups. CA1/2 and CA3 are highlighted. **c** Stereological counting revealed a reduction in the number of NeuN-positive neurons in the CA3. NeuN staining of the entorhinal cortex (EC) in control- (**d**) and Alzheimer-inoculated (**e**) groups. EC-I, EC-II, and EC-III-VI represent the different layers of the entorhinal cortex. **f** Stereological counting revealed a reduction in the number of NeuN-positive neurons in layers II and III-VI of the EC. NeuN staining of the cingulate/retrosplenial cortex (Cg/RS) in control- (**g**) and Alzheimer-inoculated (**h**) groups. **i** Stereological counting did not reveal changes of NeuN-positive neurons in the Cg/RS. Scale bars: 100 μm. *p < 0.05, Mann-Whitney test. Scatter plots display median and interquartile interval. CTRL-inoculated animals are in blue, AD1-inoculated in green and AD2-inoculated in red

Mouse lemurs were euthanized at 18 mpi, at 5.0 ± 0.2 years of age. Serial sections of the entire brains were stained with NeuN antibody and neurons counted in the hippocampus, entorhinal and cingulate/retrosplenial (RS) cortices by unbiased stereology (Fig. 4a-c). There was significant neuronal loss in the CA3 pyramidal layer of the hippocampus (*p* = 0.02) and in layers II (*p* = 0.03) and III to VI (III-VI, *p* = 0.03) of the entorhinal cortex (Fig. 4d-f). Neuronal counts in these three structures positively correlated with each other (all *p* < 0.02). Neuronal counts in the CA1/2 region of the hippocampus, layer I of the entorhinal cortex, and cingulate/retrosplenial cortex were not significantly affected by inoculation with Alzheimer’s disease brain homogenates (Fig. 4g-i). We did not detect any difference in neuronal counts in the animals inoculated with tissue homogenates from the two Alzheimer brains.

### Alzheimer’s disease brain inoculation induces β-amyloid and tau lesions

**Fig. 5:**
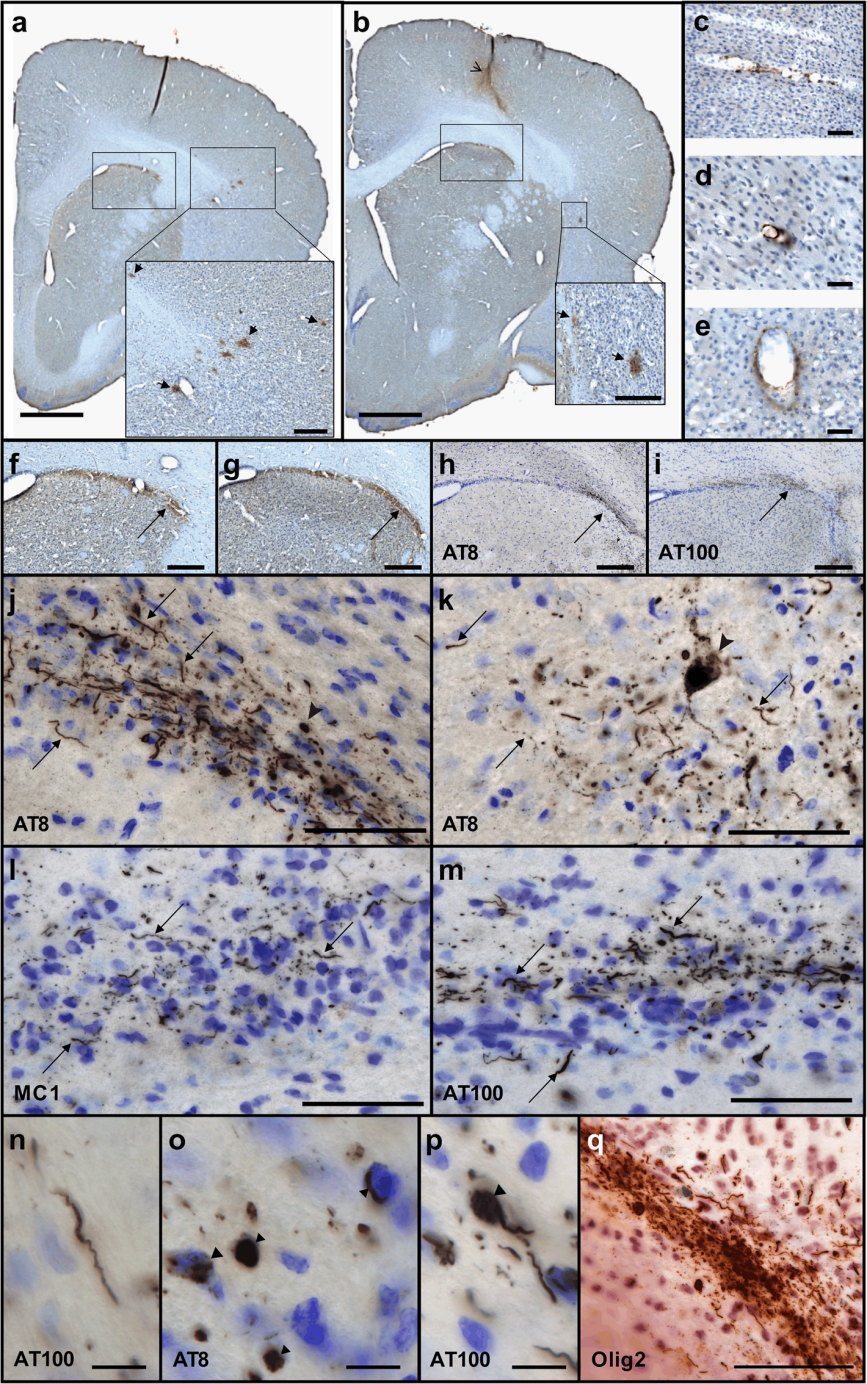
Amyloid depositions and tau inclusions in Alzheimer-inoculated mouse lemurs. **a**, **b** β-amyloid plaques (insets, arrows) revealed in two brain sections, immunostained for Aβ (4G8), separated from 1 mm in an animal from the Alzheimer-inoculated group. The inoculation site is shown with an open arrow (**b**). β-amyloid could also be detected in blood vessels (**c-e**) as well as in the parenchyma (**f-g**) close to the corpus callosum (arrows, images in **f-g** correspond to frames in **a-b**). Immunostaining for tau lesions using AT8 (**h, j, k, o**), MC1 (**l**) and AT100 (**i, m-n, p**). **h-i** frames display the same regions as the ones shown in (**f-g**): tau lesions (arrows) were detected in the same regions as Aβ. **j-n** are magnified images showing tau in neuropil threads (arrows). Intracellular tau positive structures were also detected in the form of globular positive cells (arrow), horseshoe intracellular accumulation (dotted arrow) and punctiform intracellular accumulation (arrowhead) (**o**). Rare somatodentridic inclusions (arrowhead **k, p**) as well as immunoreactive neurites with varicosities or "strings of beads" labeling (**k**: dotted arrow) were also detected. Tau stainings were counterstained with cresyl violet to identify neurons in (**h-p)**. **q** displays AT8 sections double-stained for oligodendrocytes. Tau-positive lesions were not colocalized with oligodendrocytes (AT8 (brown) and Olig2 (red)). Scale bars: 1 mm (**a-b**), 200 μm (insets in **a-b, e-i**), 50 μm (**c-e, j-m, q**), 10μm (**n-p**)

**Fig. 6:**
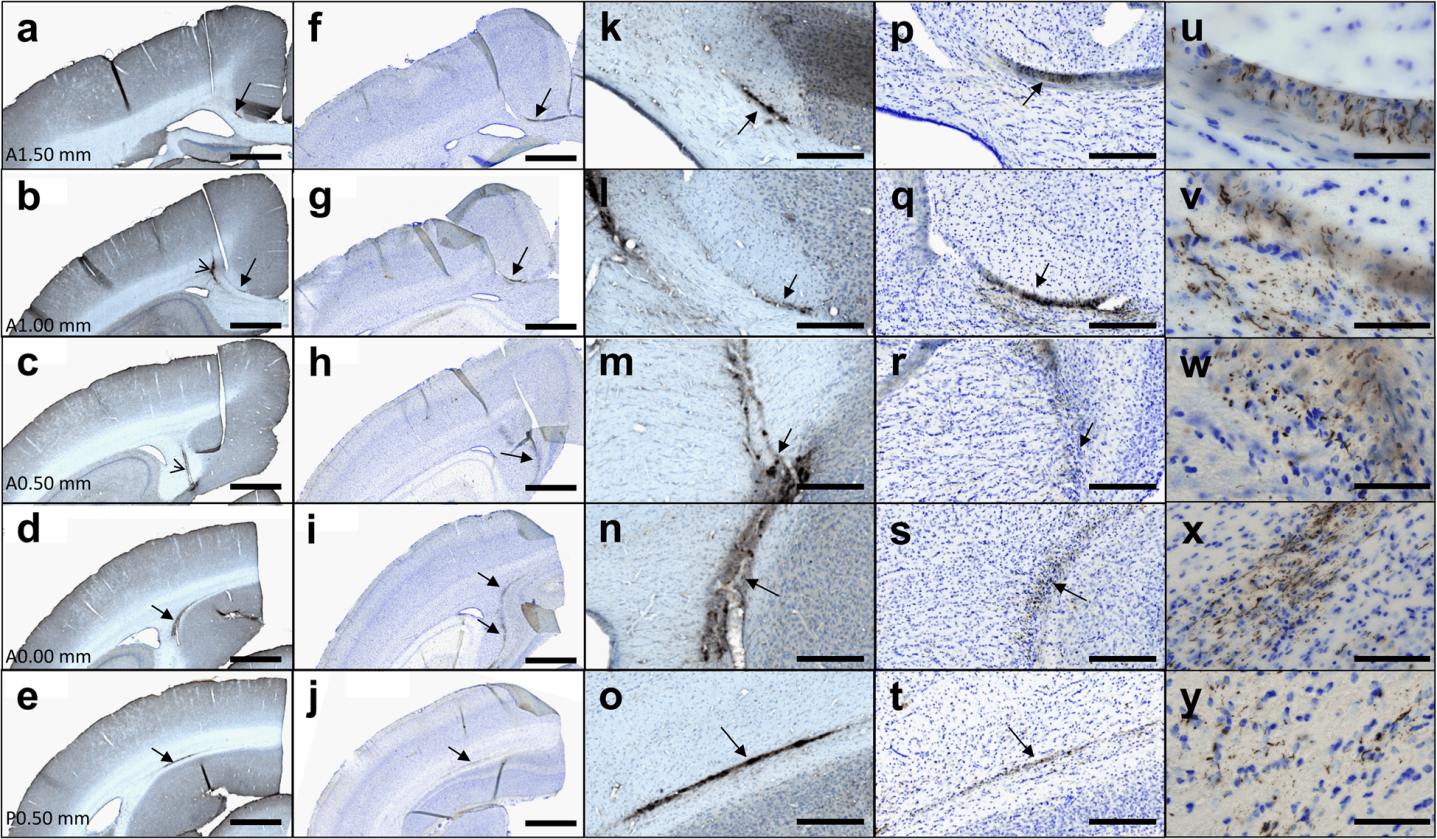
Diffusion of β-amyloid deposits and tau inclusions in Alzheimer-inoculated mouse lemurs. Immunostaining of Aβ (4G8, **a-e**, **k-o** (magnified views)) and tau (AT8, **f-j**, **p-y** (magnified views)) in 5 successive brain sections. **u-y** displays magnification of the tau-positive lesions from (**f-j**) or (**p-t**). β-amyloid and tau deposits (**a-t**) were seen exactly at the same locations (arrows). They spread from the inoculation site (open arrow in **b, c**) to regions localized one millimeter ahead and behind the inoculation site (A1.50 mm to P0.50 mm correspond to spatial references in the Bons atlas [31]). Scale bars: 1 mm (**a-j**), 200 μm (**k-t**), 50 μm (**u-y**)

We then evaluated mouse lemur brain sections for Aβ and tau pathologies (Table 1). In two out of five Alzheimer-inoculated mouse lemurs Aβ and tau deposits were detected close to the inoculation sites (Figs. 5 and 6). In the three other Alzheimer-inoculated animals Aβ deposits were also detected, but tau was not detected. Neither type of deposit was detected in the control-inoculated animals. β-amyloid deposits were found as β-amyloid plaques (Figs. 5a-b, 6) as well as bands of parenchymal β-amyloid deposits surrounding white matter tracts (Figs. 5f-g and 6) in animals inoculated with tissue homogenates from the two Alzheimer brains (AD1 and AD2). β-amyloid angiopathy was detected only in one animal inoculated with the tissue homogenate from the Alzheimer brain displaying β-amyloid angiopathy (Fig. 5c-e). Tau lesions were detected using different antibodies including AT8, MC1 and AT100 (Fig. 5h-q), and were mainly in the form of neuropil threads (Fig. 5j-n). Intracellular tau positive structures were also detected in the form of globular tau positive cells, horseshoe and punctiform tau accumulation (Fig. 5o). We also found rare somatodentridic inclusions (Fig. 5k, p) as well as immunoreactive neurites with varicosities or “strings of beads” labeling (Fig. 5k), a pattern that is considered indicative of early changes in the process of tau-related neurofibrillary degeneration [39, 40]. Tau lesions were not colocalized with oligodendrocytes (Fig. 5q), but they were always seen in regions in which β-amyloid could be detected (Fig. 5h-i and 6). The two animals displaying tau lesions were inoculated with different Alzheimer brain samples (one animal inoculated with AD1 and one animal inoculated with AD2). To further evaluate the impact of the induced tau pathology on the clinical/neuropathological outcomes, we split the Alzheimer-inoculated animals into two subgroups of tau-positive and tau-negative animals (Additional file 8: Figure S7). The two animals displaying tau lesions had the worst memory scores at 18 mpi as well as the lowest neuronal counts in the CA3 region of the hippocampus.

**Table 1:** β-amyloid and tau lesions in mouse lemurs of the Alzheimer- (AD) and control-inoculated (CTRL) groups. β-amyloid plaques or β-amyloid angiopathy (CAA) were detected only in Alzheimer-inoculated animals. Tau lesions detected in two Alzheimer-inoculated animals (AT8, MC1 and AT100 antibodies). * correspond to two animals that were euthanized at 12 months post inoculation. AD1 displayed β-amyloid angiopathy, high Aβ_1–40_ and low Aβ_1–42_ levels. AD2 did not display angiopathy and had low Aβ_1–40_ and high Aβ_1–42_ levels

Group	Animal	β-Amyloid	Tau - AT8	Tau - MC1	Tau - AT100
AD2	265B	+ (plaques)	++	++	++
AD2	260B	+ (plaques)	++	++	++
AD1	169ABC	± (plaques)	0	0	0
AD2	190IAB	± (plaques)	0	0	0
AD1	211DBA	± (CAA)	0	0	0
CTRL	189CBD	0	0	0	0
CTRL	190IC	0	0	0	0
CTRL	169ABB	0	0	0	0
CTRL	259BB*	0	0	0	0
CTRL	213ABA*	0	0	0	0

Interestingly, β-amyloid depositions and tau inclusions could be visualized in several coronal brain sections encompassing the injection sites and the levels up to 1 mm anterior and 1 mm posterior to the injection site, and up to ~ 2 mm away from the injection site in the section plane, suggesting the spreading of the lesion (Figs. 5 and 6).

We also focused on 4G8-positive intracellular staining that reflects APP/Aβ deposition in mouse lemurs [18, 19]. Intracellular staining was measured in both groups (Additional file 9: Figure S8), but there were no statistically significant differences between Alzheimer- and control-inoculated groups either in particular structures like the hippocampus (Additional file 9: Figure S8d), the caudate and putamen (not shown) or in the whole brain (Additional file 9: Figure S8h).

**Fig. 7:**
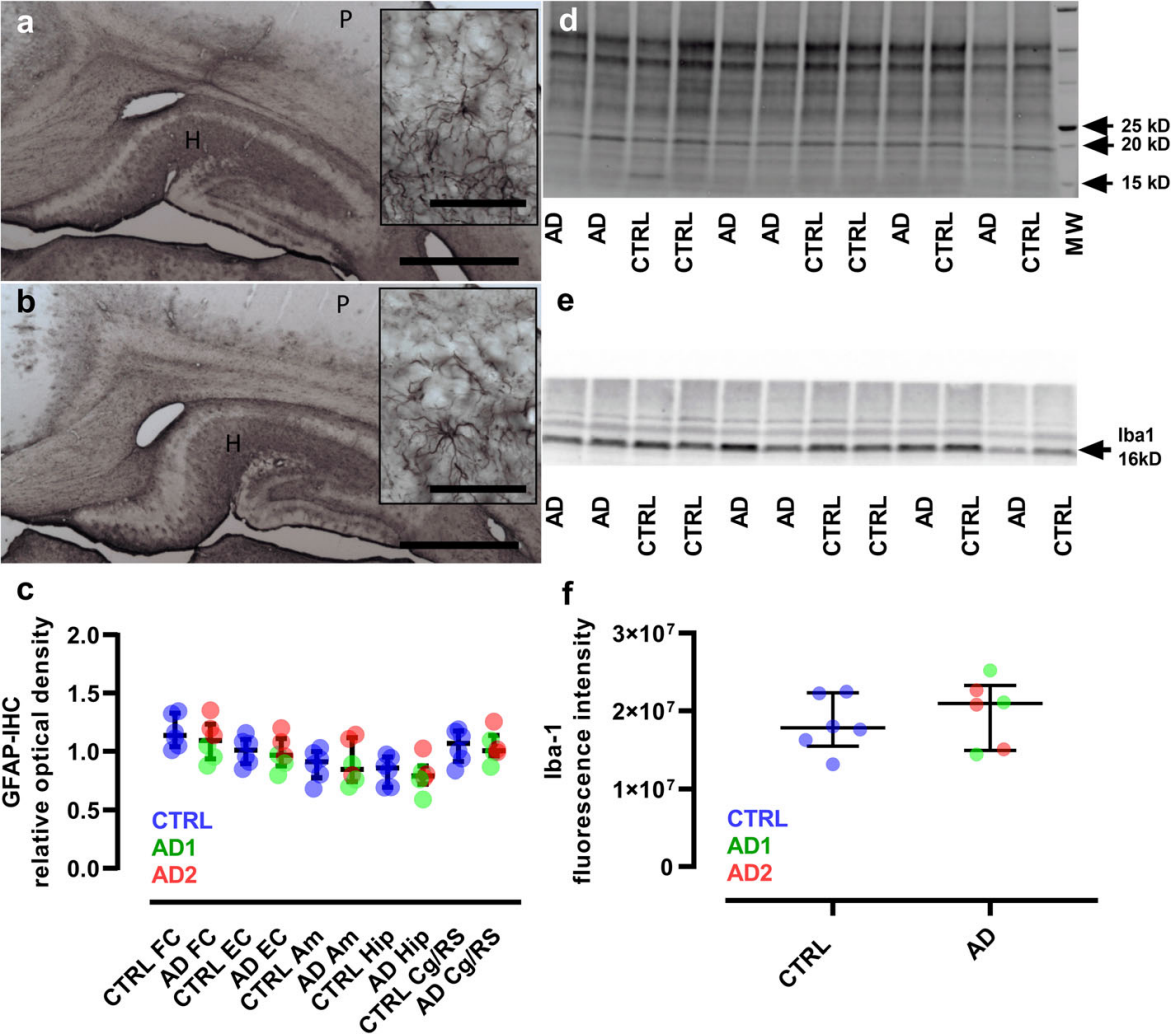
Lack of glial reactivity in inoculated lemurs. **a-b**, Immunostaining of astrocytes (GFAP) in the hippocampus (H) and parietal cortex (P) of control (**a**) and Alzheimer-inoculated (**b**) animals. Regional differences are seen including lower GFAP-immunoreactivity in the cortices which is generally found in lemurs. No qualitative difference in astrocyte morphology was detected between control- and Alzheimer-inoculated animals. Scale bars: main frame: 1 mm; inserts: 50 μm. (**c**) Quantitative evaluations of astrocyte reactivity did not provide evidence of changes in GFAP-immunoreactivity or astrocyte morphology between control- and Alzheimer-inoculated animals (Mann-Whitney tests). **d-f** Microglia reactivity was evaluated by western blot analysis (Iba1). The unstained -UV activated- blot used for total protein amount normalization is presented in (**d**), while the blot probed with Iba-1 antibody showing a specific 16kD band is displayed in (**e**). **f** Quantitative evaluations of the blots did not show any difference between Iba-1 expression in control- and Alzheimer-inoculated animals (Mann-Whitney test). Scatter plots display median and interquartile interval. CTRL-inoculated animals are in blue, AD1-inoculated in green and AD2-inoculated in red. *N* = 6 animals per group. FC: frontal cortex; EC: entorhinal cortex; Am: amygdala; Hip: hippocampus; Cg/RS: cingulate cortex/retrosplenial cortex

We did not detect obvious signs of astrocytic reactivity in any mouse lemur (Fig. 7a-b, e), and the evaluation of microglial reactivity did not reveal any difference between the Alzheimer- and control-inoculated groups (Fig. 7c-d, f). These results are consistent with the lack of differences in inflammation detected in APP/PS1_dE9_ mice between both groups (Additional file 5: Figure S4g-j).

## Discussion

This study demonstrated that inoculation of Alzheimer’s disease brain homogenates in middle-aged mouse lemurs induces alterations of long term memory and progressive loss of learning ability, modifications of neuronal activity detected by EEG, widespread and progressive cerebral atrophy, and neuronal loss in the hippocampus and entorhinal cortex. The Alzheimer’s disease brain homogenates that induced these alterations accelerated the occurrence of β-amyloid and tau lesions in transgenic mouse models of β-amyloid or tau. This result in mice is consistent with the literature [7, 8]. β-amyloid and tau lesions were also induced in mouse lemurs by inoculation with Alzheimer’s disease brain homogenates but not with the control brain homogenates. These lesions were localized close to the inoculation sites, which supports the role of the Alzheimer’s disease brain homogenates in inducing them. This is the first direct evidence of transmission of both β-amyloid and tau lesions in a non-transgenic animal. Interestingly, the lesions observed in mouse lemurs were sparse while they were more severe in transgenic mice. Thus, experimental transmission using the same Alzheimer’s disease brain homogenates leads to differential effects in different species.

Alzheimer’s disease is characterized by the presence of substantial amount of β-amyloid plaques [41, 42] in association with tau pathology [3]. In humans, the absence of these lesions precludes the diagnosis of Alzheimer’s disease. Brains of Alzheimer patients are also characterized by neuronal loss that exceeds tau pathology [2] and cerebral atrophy. Studies of the impact of experimental inoculation of Alzheimer brain homogenates in mice usually focus only on β-amyloid and tau lesions, but do not reveal consequences of inoculations on clinical signs, neuronal alterations or cerebral atrophy, despite the importance of these signs for Alzheimer’s disease. In the mouse lemurs of this study while the sparsity of the induced amyloid and tau lesions do not directly support a diagnosis for an “Alzheimer-like” pathology, the encephalopathy developed by the inoculated lemurs is clinically relevant as it was associated with cognitive alterations, widespread cerebral atrophy, modifications of neuronal activity, and neuronal loss. Further, this encephalopathy is likely related to Alzheimer pathology as it was induced by inoculation of Alzheimer brain homogenates (and not control homogenates) and because the inoculated animals presented with β-amyloid and tau lesions even if they were sparse.

The neuronal loss reported in the mouse lemurs inoculated with Alzheimer’s disease brain homogenates involved the CA3 region of the hippocampus as well as the layers II and III to VI of the entorhinal cortex. These alterations are consistent with the macroscopic atrophy detected in the hippocampus and entorhinal cortex by MRI. The Alzheimer-inoculated animals also displayed a macroscopic atrophy of the retrosplenial and posterior cingulate cortices. The CA3 region is connected with layers II and III of the entorhinal cortex by the perforant path [43], and layer II of the entorhinal cortex is connected to the retrosplenial and the posterior cingulate cortices [44]. Thus, the atrophy occurred within an organized network rather than randomly in the brain. This network connecting CA3, layer II of the entorhinal, retrosplenial and posterior cingulate cortices is strongly involved in memory for contextual information that is important for the long-term retention of a simple visual discrimination task [45, 46]. Its alteration in Alzheimer-inoculated mouse lemurs is thus consistent with their memory impairments. In addition to the neuronal loss, we found progressive impairments of neuronal activity detected first at a lower delta frequency and then at a higher theta frequency in EEG measures. This suggests a long-distance functional impact of the pathological brain homogenate on neuronal network activity.

Mouse lemurs were euthanized 18 months after inoculation of brain homogenates as they displayed clinical signs including cognitive alterations, modifications of neuronal activity detected by EEG, and cerebral atrophy. The lack of severe β-amyloidosis, tau lesions or neuroinflammation in their brains despite their clinical signs is intriguing. β-amyloid and tau lesions were detected 4 and 1 mpi in mouse models of amyloidosis and tauopathy, respectively. Thus the Alzheimer brain homogenates that we used are able to induce β-amyloid and tau lesions relatively quickly in mice. We made the choice to follow up the mouse lemurs up to 18 months after inoculation, where they reach around 5 years and are considered as middle-aged, in order to avoid age-related cognitive or neuropathological impairments. Thus, we cannot rule out the possibility that they would have developed stronger neuropathological lesions if they had lived longer. Indeed, β-amyloid and/or tau accumulation in non-human primates has been reported to take many years [11]. However, since in humans with Alzheimer’s disease the amyloid or tau lesions occur before cognitive alterations, cerebral atrophy or neuronal loss [47], we expected to detect stronger β-amyloid and/or tau lesions in lemurs that displayed clinical signs. These expectations were based on the facts that primates: i. naturally express β-amyloid or tau under normal conditions; ii. are genetically more similar to humans than transgenic mouse models of Alzheimer’s disease [48]; and iii. Can naturally display age-related cerebral atrophy associated with cognitive changes [16], all resulting in primates being relevant models to explore impact of experimental inoculation of Alzheimer brain homogenates that cannot be evaluated in mouse models. With this in mind, one interpretation of the observed presence of encephalopathy not associated with a strong inflammatory process, β-amyloid or tau deposition following Alzheimer brain homogenate intracerebral inoculation lies in research on prion diseases. Indeed, the induction of clinical signs and neuronal death in the absence of detectable pathological protein accumulation after inoculation of brain homogenates was previously reported for classical prion diseases [49]. In prion diseases, this is explained by the presence of soluble agents that are thought to be neurotoxic [50]. Further, as soluble, oligomeric, forms of β-amyloid [51] and tau [52] are known to be toxic for the brain, one possible explanation for our results is that inoculation of human Alzheimer brain homogenates led to the production of such oligomers that were toxic for neurons. The two animals presenting with aggregated tau lesions had the worst memory scores and the lowest neuronal density in CA3. Given the low density of these tau lesions, we rule out that they directly induced neuronal loss. However, they could be associated with soluble forms of tau, an entity that induces more neuronal loss than aggregated tau proteins [53]. One can however not exclude the influence of other as yet unidentified factors leading to the reported encephalopathy.

Although the evidence for cognitive and pathological alterations in Alzheimer’s brain-inoculated middle-aged animals emphasizes our findings, one limitation of the current study is that it was designed to focus on neuropathological alterations and brains were perfused with paraformaldehyde, which limited our ability to perform biochemical analysis. Future studies should include more in-depth examination of biochemical changes following Alzheimer brain inoculation. Thioflavin-T binding affinity should be used to assess the fibrillary nature of the β-amyloid and tau compounds present in animal brains [54]. β-amyloid and tau oligomers should then be evaluated by mass spectrometry [55], RT-QuiC analysis [55], immunoprecipitation experiments, and fast protein liquid chromatography [54]. Atomic force microscopy as well as a newly developed ELISA-like technique called sFIDA (Surface-Based Fluorescence Intensity Distribution Analysis Assay) should also be used to further describe the oligomers and their size [54, 56].

Another potential limitation of the study may be the small size of the animal groups that can be reached while working with primates. Using results from our studies (mean/standard deviation obtained for the different measures, proportion of animals displaying with amyloid or tau pathologies in our experimental groups) we could estimate the sample size to compare groups of control- and Alzheimer-inoculated animals assuming a significance level of 5%, a power of 80%, and two-sided tests (Additional file 1: Table S5, [57]). In mouse lemurs, small number of animals (*n* < 5) per arm are required to detect memory alterations or EEG changes after 6 mpi as well as cerebral Aβ deposition at 18 mpi. Detection of neuronal loss in the CA3 as well as in layers II and III to VI of the entorhinal cortex requires from 5 to 8 animals. These values should be compared to estimations of sample sizes in mice that require from 1 to 8 mice to assess tau or amyloid deposition. They show that the number of mouse lemurs required to obtain scientific results is in the same range as the one required for transgenic rodents. Mouse lemurs however provide new types of information as they can spontaneously display amyloid or tau lesions on a wild-type/primate genetic background as well as neuronal loss associated to clinical outcomes. They can be ideal models to assess the impact of various amyloid/tau strains on disease occurrence or the role of oligomers on clinical outcomes in primates.

In conclusion, our results indicate that Alzheimer’s disease brain homogenate inoculation induces an encephalopathy characterized by neuronal loss, progressive atrophy, neuronal activity alterations and cognitive impairments as well as sparse β-amyloid and tau depositions. The clinical signs can be explained by the neuronal loss, cerebral atrophy and neuronal networks dysfunction. Tau lesions may be a strong determinant, but not the only one, in the induction of the neuronal loss and clinical outcome. Further studies are necessary to evaluate the nature of relationships between the different lesions induced by Alzheimer’s disease brain homogenate inoculation and to assess the mechanisms leading to encephalopathy induced by these inoculations.

## Additional files

Additional file 1: **Table S1** Human brain sample characteristics and staging. **Table S2** Schedule of the experimental protocol. **Table S3** Sampling parameters for stereological counting of NeuN-positive neurons. **Table S4** Brain regions with gray matter loss in the Alzheimer's disease-inoculated group relative to the control-inoculated group. **Table S5** Estimated sample size to compare control and Alzheimer-inoculated mice and mouse lemurs assuming a significance level of 5%, a power of 80%, and two-sided tests. (DOCX 46 kb)

Additional file 2: **Figure S1** Cognitive test in mouse lemurs. (TIF 5572 kb)

Additional file 3: **Figure S2** Characterization of human brain samples and homogenates. (TIF 19464 kb)

Additional file 4: **Figure S3** Prion protein examination in human brain samples. (TIF 9854 kb)

Additional file 5: **Figure S4** Aβ and Tau pathology in mice after inoculation with human brain homogenates. (TIF 26552 kb)

Additional file 6: **Figure S5** Correlation between cognitive abilities and EEG delta frequency. (TIF 4153 kb)

Additional file 7: **Figure S6** Time-dependent evolution of cerebral atrophy in inoculated lemurs. (TIF 19870 kb)

Additional file 8: **Figure S7** Impact of tau pathology on memory and neuronal loss. (TIF 8800 kb)

Additional file 9: **Figure S8** Similar level of intracellular 4G8-positive structures in Alzheimer’s disease and control-inoculated lemurs. (TIF 21279 kb)

## Data Availability

The datasets during and/or analysed during the current study available from the corresponding author on reasonable request.
