# Supplementary material for: Encephalopathy induced by Alzheimer brain inoculation in a non-human primate
Source: Acta Neuropathol Commun. 2019 Sep 4;7:126. doi: 10.1186/s40478-019-0771-x (PMC6724379; doi:10.1186/s40478-019-0771-x)
Supplement: Supplementary file 1 — Table S1 Human brain sample characteristics and staging. Table S2 Schedule of the experimental protocol. Table S3 Sampling parameters for stereological counting of NeuN-positive neurons. Table S4 Brain regions with gray matter loss in the Alzheimer's disease-inoculated group relative to the control-inoculated group. Table S5 Estimated sample size to compare control and Alzheimer-inoculated mice and mouse lemurs assuming a significance level of 5%, a power of 80%, and two-sided tests. (DOCX 46 kb) [file 40478_2019_771_MOESM1_ESM.docx]

**Encephalopathy induced by Alzheimer brain inoculation**

**in a non-human primate**

**Supplementary Tables**

**Charlotte Gary^1,2^, Suzanne Lam^1,2,$^, Anne-Sophie Hérard^1,2,$^, James E. Koch^1,2,3^, Fanny Petit^1,2^, Pauline Gipchtein^1,2^, Stephen J. Sawiak^4,5^, Raphaëlle Caillierez^6^, Sabiha Eddarkaoui^6^, Morvane Colin^6^, Fabienne Aujard^7^, Jean-Philippe Deslys^8^, French Neuropathology Network^9,^, Emmanuel Brouillet^1,2^, Luc Buée^6^, Emmanuel E. Comoy^8^, Fabien Pifferi^7,#^, Jean-Luc Picq^1,2,10,#^, Marc Dhenain^1,2,*^**

^1^ CNRS, UMR 9199, Neurodegenerative Diseases Laboratory, Fontenay-aux-Roses, France ; ^2^ CEA, DRF, MIRCen, Fontenay-aux-Roses, France; ^3^ University of Wisconsin Oshkosh, Oshkosh, WI 54901, USA; ^4^ Wolfson Brain Imaging Centre, University of Cambridge, Cambridge, UK; ^5^ Behavioural and Clinical Neuroscience Institute, University of Cambridge, Cambridge, UK; ^6^ Université de Lille, Inserm, CHU-Lille, UMR-S1172, Alzheimer & Tauopathies, Lille, France; ^7^ UMR7179 CNRS-MNHN, MECADEV, Brunoy, France; ^8^ CEA, DRF, SEPIA, , Fontenay-aux-Roses, France; ^9^ GIE Neuro-CEB/Neuropathologist Network, Hôpital de la Pitié-Salpêtrière, Paris, France; ^10^ Laboratoire de Psychopathologie et de Neuropsychologie, Université Paris 8, St-Denis, France.

^$, #^ These authors contributed equally to this work.

Corresponding author: Marc Dhenain, DVM, PhD

Commissariat à l’Energie Atomique et aux Energies Alternatives (CEA), Direction de la Recherche Fondamentale (DRF), Institut François Jacob, MIRCen, 18 Route du Panorama, F-92265 Fontenay-aux-Roses, France

## E-mail: [marc.dhenain@cea.fr](mailto:marc.dhenain@cea.fr)

| \| Patient \| Braak stage \| Thal phase \| Age (years) \| Post-mortem delay (hours) \| Immunohistochemistry \| \| --- \| --- \| --- \| --- \| --- \| --- \| \| AD1 \| VI \| 5 \| 76 \| 10 \| Tau-positive (AT8, Innogenetic, 1/500)  Aβ-positive, including amyloid angiopathy (6F3D, Dako, 1/200)  Alpha-synuclein-negative (LB509, Zymed, 1/250) \| \| AD2 \| VI \| 4 \| 83 \| 21 \| Tau-positive (AT8, Innogenetic, 1/500)  Aβ-positive, without amyloid angiopathy (6F3D, Dako, 1/200)  Alpha-synuclein negative (LB509, Zymed, 1/250) \| \| CTRL \| 0 \| 0 \| 69 \| 6 \| Tau-negative (AT8, Innogenetic, 1/500)  Aβ-negative (6F3D, Dako, 1/200)  Alpha-synuclein-negative (LB509, Zymed, 1/250) \| |
| --- | --- | --- | --- | --- | --- | --- | --- | --- | --- | --- | --- | --- | --- | --- | --- | --- | --- | --- | --- | --- | --- | --- | --- | --- |
| **Supplementary Table 1 Human brain sample characteristics and staging.** Human parietal cortex samples were obtained from two Alzheimer’s patients (AD1 and AD2) and one control (CTRL) individual. They were tested for several pathologies by the French reference laboratory (C. Duyckaerts, Pitié-Salpêtrière hospital, Paris, France). Only the Alzheimer brains presented Alzheimer lesions. No other pathologies were detected in the brains. |

| Experimental protocol | Before inoculation | 3 mpi | 6 mpi | 9 mpi | 12 mpi | 15 mpi | 18 mpi | |
| --- | --- | --- | --- | --- | --- | --- | --- | --- |
| Rotarod test | X |  | X |  | X |  | X | |
| Learning tasks | X |  | X |  | X |  | X | |
| Long-term memory tasks |  |  | X |  | X |  | X | |
| Electroencephalography | X |  | X |  | X |  |  | |
| MRI and atrophy evaluation | X | X | X | X | X | X | X | |
| Immunohistochemistry |  |  |  |  |  |  | X | |
| Stereological evaluation of neuronal loss (NeuN) |  |  |  |  |  |  | X | |
| Evaluation of amyloid (4G8) and tau (AT8, MC1, AT100) lesions |  |  |  |  |  |  | X | |
| **Supplementary Table 2 Schedule of the experimental protocol.** mpi: months post-inoculation. MRI: magnetic resonance imaging. | | | | | | | |  |

| Brain regions | SSF | NES | ACF (µm²) | x-y steps (µm) | ASF | TSF-µm mean±SEM | CE mean±SEM | Sampled cells per animal |
| --- | --- | --- | --- | --- | --- | --- | --- | --- |
| CA1/2 | 1/10 | 7-10 | 30*30 | 50-50 | 0.36 | 11.63±0.03 | 0.037±0.002 | 430-700 |
| CA3 | 1/10 | 5-7 | 30*30 | 50-50 | 0.36 | 11.63±0.03 | 0.064±0.002 | 485-760 |
| EC-I | 1/10 | 6 | 100*100 | 150-150 | 0.44 | 12.21±0.10 | 0.067±0.001 | 90-170 |
| EC-II | 1/10 | 6 | 30*30 | 75-75 | 0.16 | 12.21±0.10 | 0.073±0.000 | 400-530 |
| EC-III-VI | 1/10 | 6 | 50*50 | 150-150 | 0.11 | 12.21±0.10 | 0.064±0.000 | 650-800 |
| Cing/RS | 1/10 | 5-8 | 50*50 | 150-150 | 0.11 | 11.59±0.07 | 0.056±0.001 | 1600-2500 |

**Supplementary Table 3 Sampling parameters for stereological counting of NeuN-positive neurons.** SSF: Section sampling fraction; NES: Number of evaluated sections; ACF: Area of counting frames; x-y steps: x-y steps between each counting frame; ASF: Area of the sampling fractions; TSF: Thickness of the sampling fraction; CE: Coefficient of error; CA1/2: CA1/2 region of the hippocampus; CA3: CA3 region of the hippocampus; EC-1, EC-2, EC-III-VI: Layers I, II, III-VI of the entorhinal cortex; Cing/RS: Cingulate cortex/retrosplenial cortex.

| **Brain regions** | **Cluster size (voxels)** | **Peak p-value FDR-corrected** | | **Peak T value** |
| --- | --- | --- | --- | --- |
| Entorhinal cortex, amygdala, hippocampus and inferior temporal cortex | 1562 | *** | 0.0004 | 6.3952 |
| Cingulate and retrosplenial cortices | 1342 | *** | 0.0004 | 6.3777 |
| Diagonal band of Broca, fornix and nucleus and stria terminalis | 184 | ** | 0.0013 | 5.7422 |
| Ventral hippocampus | 103 | * | 0.0109 | 4.2790 |
| Caudate nucleus | 70 | ** | 0.0059 | 4.8479 |
| Entorhinal cortex | 59 | * | 0.0117 | 4.2231 |
| Lateral temporal cortex | 55 | ** | 0.0062 | 4.7998 |
| Peri-third ventricule area | 52 | * | 0.0112 | 4.2488 |
| Inferior temporal cortex | 47 | * | 0.0175 | 3.8968 |
| Fornix and stria terminalis | 33 | ** | 0.0061 | 4.8140 |
| Parietal cortex | 31 | ** | 0.0045 | 5.0162 |
| Amygdala | 30 | * | 0.0102 | 4.4005 |
| Lateral temporal cortex | 23 | * | 0.0221 | 3.6795 |
| Amygdala | 22 | * | 0.0267 | 3.4984 |
| Parietal cortex | 18 | * | 0.0227 | 3.6538 |
| Amygdala | 18 | * | 0.0255 | 3.5305 |
| Inferior temporal cortex | 17 | * | 0.0221 | 36785 |
| Caudate nucleus | 16 | ** | 0.0067 | 4.7113 |
| Fornix | 15 | * | 0.0172 | 3.9105 |
| Inferior temporal cortex | 15 | * | 0.0193 | 3.7971 |
| Caudate nucleus | 14 | * | 0.0233 | 3.6270 |
| Inferior temporal cortex | 13 | * | 0.0234 | 3.6142 |
| Inferior temporal cortex | 13 | * | 0.0140 | 4.0705 |
| Entorhinal cortex | 11 | * | 0.0135 | 4.1200 |
| Parietal cortex | 10 | * | 0.0108 | 4.3009 |
| Amygdala | 10 | * | 0.0255 | 3.5285 |

**Supplementary Table 4** **Brain regions with gray matter loss in the Alzheimer's disease-inoculated group relative to the control-inoculated group**. N = 6 animals per group at 0, 3, 6, 9 mpi and n = 6 and 4 in the Alzheimer- and control-inoculated groups, respectively at 12, 15, and 18 mpi. *p < 0.05; **p < 0.01; ***p < 0.001.

| Species | Measure | mpi | Sample size |
| --- | --- | --- | --- |
| Tau30^+/+^ mice | Tau deposition in the soma of hippocampal neurons | 1 | 1 |
| APP/PS1dE9 mice | Aβ deposition in the hippocampus | 4 | 8 |
| Mouse lemurs | Learning task D2 | 6 | 249 |
| Mouse lemurs | Learning task D3 | 12 | 10 |
| Mouse lemurs | Learning task D4 | 18 | 13 |
| Mouse lemurs | Memory Task D1r | 6 | 1 |
| Mouse lemurs | Memory task D2r | 12 | 3 |
| Mouse lemurs | Memory task D3r | 18 | 2 |
| Mouse lemurs | EEG Delta 6 mpi | 6 | 3 |
| Mouse lemurs | EEG Theta 6 mpi | 6 | 4 |
| Mouse lemurs | EEG Delta 12 mpi | 12 | 4 |
| Mouse lemurs | EEG Theta 12 mpi | 12 | 9 |
| Mouse lemurs | EEG Alpha 12 mpi | 12 | 3 |
| Mouse lemurs | EEG Sigma 12 mpi | 12 | 3 |
| Mouse lemurs | EEG Beta 12 mpi | 12 | 3 |
| Mouse lemurs | NeuN-CA3 | 18 | 5 |
| Mouse lemurs | NeuN-CA1-2 | 18 | 1067 |
| Mouse lemurs | NeuN-EC-III-VI | 18 | 8 |
| Mouse lemurs | NeuN-EC-II | 18 | 7 |
| Mouse lemurs | NeuN-EC-I | 18 | 52 |
| Mouse lemurs | NeuN-Cg/RS | 18 | 46 |
| Mouse lemurs | Aβ deposition in the brain | 18 | 3 |
| Mouse lemurs | CAA in the brain | 18 | 35 |
| Mouse lemurs | Tau deposition in the brain | 18 | 15 |

**Suppl. Table 5** Estimated sample size to compare control and Alzheimer-inoculated mice and mouse lemurs assuming a significance level of 5%, a power of 80%, and two-sided tests. The different effects were classified according to species (Tau30+/+ mice, APP/PS1dE9 mice or mouse lemurs). mpi represents the time post inoculation for the measure. Sample size represents the number of animal per experimental arm.
